# Noninvasive Thrombus Imaging in Patients With Ischemic Stroke

**DOI:** 10.1161/CIRCULATIONAHA.126.080420

**Published:** 2026-09-01

**Authors:** Beth Whittington, Craig Balmforth, Alessandro Giaj Levra, Laura Clark, Neil Craig, Audrey White, Krithika Loganath, Evangelos Tzolos, Allison Winarski, Rachael O. Forsythe, Michael McDermott, Tariq Ramtoola, Phyo H. Khaing, Sphamandla Ntshangase, Mark G. Macaskill, Stefan Veizades, Mairi Brittan, Tim Clark, Michelle Rooney, Christophe Lucatelli, Piotr J. Slomka, Norman Koglin, Andrew W. Stephens, Iris Hardewig, Edwin J.R. van Beek, Marc R. Dweck, Joanna Wardlaw, Michelle C. Williams, William N. Whiteley, David E. Newby

**Affiliations:** 1 British Heart Foundation Center of Research Excellence (B.W., C.B., A.G.L., L.C., N.C., A. White, K.L., E.T., A. Winarski, R.O.F., M.M., T.R., P.H.K., S.N., M.G.M., S.V., M.B., E.J.R.v.B., M.R.D., M.C.W., D.E.N.), University of Edinburgh, Edinburgh, United Kingdom.; 2 Institute of Neuroscience and Cardiovascular Research (J.W., W.N.W.), University of Edinburgh, Edinburgh, United Kingdom.; 3 Edinburgh Imaging, Queen’s Medical Research Institute, Edinburgh, United Kingdom (T.C., M.R., C.L.).; 4 Cedars-Sinai Medical Center 22494 , Los Angeles, CA (P.J.S.).; 5 Lantheus Germany GmbH, Berlin, Germany (N.K., A.W.S., I.H.).

**Keywords:** atherosclerosis, platelets, positron emission tomography, stroke, thrombosis

## Abstract

**BACKGROUND:**

Determining the source of thromboembolism in patients with acute ischemic stroke remains challenging because current diagnostic approaches rely largely on circumstantial and inferential associations rather than direct identification of the cause of stroke. Fluorine-18 GP1 ([^18^F]GP1) is a novel radiotracer that binds with high affinity and specificity to activated glycoprotein IIb/IIIa receptors on activated platelets. We aimed to determine whether hybrid [^18^F]GP1 positron emission tomography (PET) and computed tomography angiography could identify in vivo cardiovascular thromboembolism in patients with acute ischemic stroke and provide a mechanism-based assessment of stroke etiology.

**METHODS:**

In a single-center prospective observational cohort study, 100 patients presenting with acute ischemic stroke underwent hybrid [^18^F]GP1 PET/computed tomography angiography within 21 days of symptom onset in addition to standard-of-care stroke investigations. Stroke etiology was classified using the causative classification system by an expert stroke physician blinded to [^18^F]GP1 PET findings. In parallel, [^18^F]GP1 PET/computed tomography angiography images were analyzed independently by readers who were blinded to all clinical data and causative classification system classification. We assessed whether [^18^F]GP1 PET/computed tomography angiography could identify the source of in vivo cardiovascular thromboembolism, influence stroke classification, and predict recurrent cerebrovascular events.

**RESULTS:**

Cardiovascular [^18^F]GP1 uptake indicative of thrombosis was identified in nearly two-thirds (n=63) of participants, changing stroke classification in over a quarter (n=26) of patients. In those with stroke of undetermined cause, the source of thrombus was identified in 18 of 41 (44%) participants, which included nonstenotic carotid atherothrombosis, native cardiac valve thrombosis, and paradoxical thromboembolism. During a median follow-up of 613 (interquartile interval, 251–788) days, recurrent stroke or transient ischemic attack occurred in 14 patients, 13 (93%) of whom had cardiovascular [^18^F]GP1 uptake at baseline. Cardiovascular [^18^F]GP1 uptake was associated with a 10.4-fold increased risk of recurrent events (95% CI, 1.35–79.40; *P*=0.024).

**CONCLUSIONS:**

Noninvasive thrombosis imaging is a novel technique that enables direct in vivo identification of thrombus formation and frequently reveals the source of thromboembolism in patients with acute ischemic stroke. This approach represents a shift from inference-based to mechanism-based stroke classification with potential implications for targeted prevention strategies and better patient outcomes.

**REGISTRATION:**

URL: https://www.clinicaltrials.gov; Unique Identifier: NCT05636748.

**Clinical Perspective**

**What Is New?**

- Total-body fluorine-18 GP1 positron emission tomography and computed tomography angiography identify active thrombosis across multiple cardiovascular territories in patients with acute ischemic stroke.
- Fluorine-18 GP1 uptake, indicative of cardiovascular thrombosis, is associated with a 10-fold increased risk of recurrent stroke independent of age, sex, and traditional cardiovascular risk factors.

**What Are the Clinical Implications?**

- Molecular thrombus imaging can reclassify stroke etiology in a quarter of all patients, including nearly half of patients with stroke of undetermined cause.
- Cardiovascular thromboembolism is the dominant cause of recurrent ischemic stroke.
- Molecular thrombus imaging could transform the investigation and treatment of ischemic stroke by shifting from current inference-based management to mechanism-based diagnosis, clinical decision-making, and pathways of care, which has the potential to improve clinical outcomes.

**Editorial, see p 1441**

Stroke remains the second leading cause of death and the third leading cause of death and disability with 160 million disability-adjusted life-years lost globally.^1–3^ The incidence of stroke has risen substantially worldwide over the last 30 years, with the estimated global cost nearly $900 billion (0.66% of the global gross domestic product) per year. This is projected to double by 2050.^3^ In individuals who have recently had a minor stroke or transient ischemic attack, the risk of recurrent stroke over 10 years is up to 20%, with a third of events recurring in the first year.^4^ Thus, the prevention of recurrent stroke is a key priority and unmet need. Improved methods for the identification of sources of thrombus could allow interventions to be targeted to the mechanisms of stroke and thereby prevent recurrent stroke.

Acute ischemic strokes are predominantly caused by thromboembolism. To identify potential sources of thromboembolism, patients undergo multiple assessments that include cardiac rhythm monitoring, carotid ultrasonography, transthoracic and transesophageal echocardiography, magnetic resonance imaging, and computed tomography angiography. This is a time-consuming and resource-intensive process that often fails to identify the thromboembolism source.^5^ Indeed, 39% of all strokes have an undetermined etiology,^6^ and in those with a defined cause, the etiology is often circumstantial or inferential, such as the presence of atrial fibrillation or carotid atheroma with over 50% stenosis of the ipsilateral internal carotid artery.

We previously evaluated an emerging fluorine-18–labeled radiotracer, fluorine-18 GP1 ([^18^F]GP1), for the noninvasive in vivo identification of acute venous and arterial thrombosis across a range of cardiovascular diseases.^7–11^ [^18^F]GP1 is a modification of the small molecule elarofiban, a glycoprotein IIb/IIIa receptor antagonist.^12^ The expression of glycoprotein IIb/IIIa is platelet specific, and following platelet activation, the receptor undergoes allosteric activation, resulting in a conformational change from a low-affinity to a high-affinity receptor for its natural ligand fibrin, a key step in platelet aggregation and acute thrombus formation.^13,14^ [^18^F]GP1 binds with high affinity and specificity to the activated glycoprotein IIb/IIIa receptors on activated platelets, enhancing its ability to detect acute platelet-rich thrombi. In a recent pilot study, we demonstrated that [^18^F]GP1 positron emission tomography (PET) and computed tomography (CT) angiography could detect in vivo thrombosis in the carotid arteries and brain of 11 patients with acute ischemic stroke or transient ischemic attack.^7^ Based on these promising preliminary findings, we aimed to determine whether [^18^F]GP1 PET/CT angiography could identify in vivo cardiovascular thromboembolism, influence stroke classification, and be associated with recurrent cerebrovascular events across a broad spectrum of patients with acute ischemic stroke.

## Methods

### Data Availability

The data that support the findings of this study are available from the corresponding author upon reasonable request.

### Study Design and Study Population

This was a prospective single-center observational cohort study investigating the role and origin of thromboembolism in acute ischemic stroke (NCT05636748). From March 2023 to June 2025, we prospectively screened all adults presenting to the Royal Infirmary of Edinburgh with a clinical diagnosis of ischemic stroke. Patients were approached consecutively if they met the predefined inclusion criteria and none of the predefined exclusion criteria (Figure S1). We recruited patients over the age of 18 years who presented with acute ischemic stroke and had imaging evidence of recent cerebral infarction on either CT or magnetic resonance imaging (MRI). Exclusion criteria included hemorrhagic stroke, inability to give informed consent, inability to tolerate the scanning protocol, estimated glomerular filtration rate <30 mL/min per 1.73 m^2^, allergy to iodinated contrast media, and women who were pregnant or breastfeeding.

Enrolled participants all underwent clinical assessment, including cardiovascular and neurological examination, 12-lead ECG, 3- to 5-day ECG monitoring, bubble-contrast echocardiography, carotid Doppler ultrasound, and brain imaging with either noncontrast CT or MRI.

The study was approved by the West of Scotland Research Ethics Committee (21/WS/0165), conducted in accordance with the Declaration of Helsinki, and undertaken with the written informed consent of all participants.

### [^18^F]GP1 PET and CT Angiography

All participants underwent hybrid [^18^F]GP1 PET/CT angiography within 21 days of stroke symptom onset. Following intravenous injection of a target dose of 250 MBq [^18^F]GP1, total-body PET imaging was performed with electrocardiographic gating. Contrast-enhanced CT angiography of the cardiac, carotid, cerebral, and lower limb vasculature was performed immediately following PET acquisition. Detailed acquisition and reconstruction parameters are provided in the Supplemental Methods.

### CT Angiography and Venography Image Analysis

Contrast-enhanced CT angiograms and lower-limb venograms were analyzed by expert radiologists (M.C.W. and E.J.R.v.B.) blinded to the results of the [^18^F]GP1 PET scan. Arterial territories, including the arch of the aorta and its branches, carotid and vertebral arteries, as well as the intracranial arteries, were assessed for the presence of atherosclerotic plaque; plaque characteristics, including high-risk features; and the degree of carotid artery stenosis according to the NASCET (North American Symptomatic Carotid Endarterectomy Trial) criteria.^15^ Venous imaging was evaluated for the presence of pelvic and lower-limb thrombosis.

### Stroke Classification

An expert stroke physician (W.W.) reviewed the clinical information for each study participant alongside their standard-of-care investigations (CT or MRI), 12-lead ECG, prolonged ECG monitoring, bubble-contrast echocardiography, and contrast CT angiography. The most likely cause of stroke was determined using the causative classification system (CCS) for stroke.^16^ CCS classification was performed before revealing the qualitative results of the [^18^F]GP1 PET/CT scan. In parallel, [^18^F]GP1 PET images were analyzed independently by readers who were blinded to all clinical data and CCS classification. The [^18^F]GP1 PET/CT scan findings were then provided to the expert stroke physician, who determined whether these findings changed the CCS classification for stroke. For causal attribution, we considered that any [^18^F]GP1 uptake was clinically relevant and was used to inform potential CCS reclassification. Only carotid or vertebral artery [^18^F]GP1 uptake ipsilateral to the cerebral infarct territory was considered clinically relevant and permitted to inform potential CCS reclassification. Contralateral arterial uptake was recorded but did not influence CCS assignment. In cases of multiple sites or bilateral carotid [^18^F]GP1 uptake, the PET reader remained blinded to the side of stroke symptoms at the time of image review to avoid laterality bias in the qualitative assessment of the PET signal. All sites of [^18^F]GP1 uptake were provided to the stroke classifier, along with their anatomical location and relation to the infarct territory. Attribution was based on anatomical plausibility and established stroke mechanisms, and a prespecified blinded workflow minimized bias in both lesion identification and causal attribution.

### [^18^F]GP1 PET Image Analysis

Images were analyzed using dedicated software (FusionQuant V1.21.0421, Cedars Sinai Medical Center, Los Angeles, CA) as described previously.^9,10^ Before analysis, reconstructed PET images were fused with CT angiograms using rigid translation of PET images and alignment with points of reference, such as the bone marrow of the cervical vertebra and the blood pool of jugular veins and great vessels for carotid and brain image analysis, as described previously.^7,17^ For cardiac analysis, coregistration was performed using a rigid translation of PET images using 5 anatomical points of reference (the blood pool of the left ventricle, the right ventricle, the great vessels and bone marrow of vertebra, and the sternum), as described previously.^9,10,18^

Qualitative [^18^F]GP1 uptake was assessed by consultant radiologists (M.C.W., E.J.R.v.B., and J.M.W.) who were blinded to all clinical data and CCS classification. Qualitative assessment classified [^18^F]GP1 uptake as present or absent based on focal tracer accumulation above background activity.

Quantitative assessments of PET uptake within the brain, carotid arteries, cardiac valves, cardiac chambers, and lower-limb vasculature were assessed for [^18^F]GP1 uptake by 2 independent readers (C.B. and A.G.L.) who were blinded to all clinical data and CCS classification. Quantitative uptake values were assessed within spheres of interest using maximum standardized uptake values, mean standardized uptake values, maximum target-to-background ratio, and mean target-to-background ratio. Quantitative thresholds were used as supportive evidence for visual qualitative assessments. For details of regional imaging analyses, see Supplemental Methods.

### Ex Vivo Carotid Plaque Analysis

Carotid atheroma specimens were obtained from patients (n=3) undergoing carotid endarterectomy. All specimens were derived from participants who had undergone preoperative [^18^F]GP1 PET/CT imaging, enabling direct image-matched comparisons between in vivo PET signal and ex vivo tissue analysis. Immediately following surgical excision, specimens were flash frozen in liquid nitrogen and stored at −80°C before analysis.

For histological assessment, frozen tissue samples were sectioned and subsequently formalin fixed and paraffin embedded. Hematoxylin-eosin staining was performed on 10-µm sections to assess plaque morphology. Immunohistochemistry for platelet identification was performed using a rabbit monoclonal anti-CD41 antibody (EPR4330, Abcam, lot GR3259786) at a dilution of 1:100. Staining was performed using an automated Leica Bond Rx system with Bond Epitope Retrieval Solution 1 (pH 6, catalog No. AR9961) and Bond Polymer Refine Red Detection (catalog No. DS9390). Omission of the primary antibody was used as a negative control.

Autoradiography was performed on adjacent tissue sections to assess [^18^F]GP1 binding. Frozen sections (5 µm) were incubated with 1 nM [^18^F]GP1 for 60 minutes at room temperature, with and without coincubation with tirofiban (10 µM), to assess nonspecific binding. Following incubation, sections were washed twice in phosphate-buffered saline and once in deionized water, dried, and prepared for imaging. Autoradiography images were acquired over 60 minutes using a microimager (Biospace Lab, Nesles-la-Vallée, France).

Quantitative analysis was performed using software developed in house (MEDgical). Regions of interest were manually delineated over the entire tissue section on coregistered optical images. Tracer uptake was expressed as counts per minute, corrected for radioactive decay to the start of tracer incubation and normalized to the surface area of the region of interest (counts per minute per square centimeter).

### Clinical Follow-Up

Participants were followed up for outcomes of clinically diagnosed recurrent stroke or transient ischemic attack following their incident stroke event. Outcome information was obtained from the local and national health care record systems, incorporating primary and secondary care records and the Scottish Stroke Care Audit database. Complete follow-up was achieved for 100% of study participants. During this data collection, study investigators were blinded to the PET/CT data.

### Statistical Analysis

Statistical analyses were performed using RStudio version 2023.06.1 (R Foundation for Statistical Computing, Vienna, Austria).

Categorical variables are presented as counts (percentages). Continuous variables were assessed for normality using visual inspection of histograms and Q-Q plots and are presented as mean±SD where normally distributed and median (interquartile interval) where non-normally distributed. Comparisons between groups were performed using the independent-sample *t* test for normally distributed variables or the Mann-Whitney U test for non-normally distributed variables. Comparisons across more than 2 groups were performed using analysis of variance or the Kruskal-Wallis test, as appropriate. Categorical variables were compared using the χ^2^ test or Fisher exact test, as appropriate.

For carotid analyses, the unit of analysis was the individual artery. Associations between plaque characteristics and [^18^F]GP1 uptake (treated as a binary outcome on the basis of visual PET positivity) were assessed using logistic regression with robust standard errors clustered by patient to account for the correlated nature of bilateral carotid artery data. Results are reported as odds ratios and 95% CIs.

Time-to-event analyses were performed using Kaplan-Meier methods, with time to first recurrent stroke or transient ischemic attack as the outcome; multiple recurrent events per patient were not modeled. Groups were compared using the log-rank test, and Cox proportional hazards regression was used to assess associations between [^18^F]GP1 uptake and recurrent events. The proportional hazards assumption was assessed using Schoenfeld residuals. No deaths occurred during follow-up, and competing risk analysis was not performed. Variables included in multivariable models were selected a priori on the basis of clinical relevance and established vascular risk factors. Given the relatively small number of outcome events, the number of variables in each model was limited to reduce the risk of overfitting. Age and sex were included in a separate sensitivity model.

All statistical tests were 2 sided, and *P*<0.05 was considered statistically significant.

## Results

### Study Population

Following screening and eligibility assessment (Figure S1), 100 patients (median age, 65 years [interquartile interval, 54–73], 68% men) with acute ischemic stroke underwent [^18^F]GP1 PET/CT angiography within a median of 14 [interquartile interval, 10–17] days from symptom onset. Using the CCS for stroke, 41 strokes were due to an undetermined etiology, 33 were due to cardioembolism, 9 were due to large-artery atherosclerosis, and 14 were due to small-artery occlusion (Table 1). The remaining 3 were classified as “other uncommon cause” and were secondary to carotid arterial dissections.

**Table 1.**
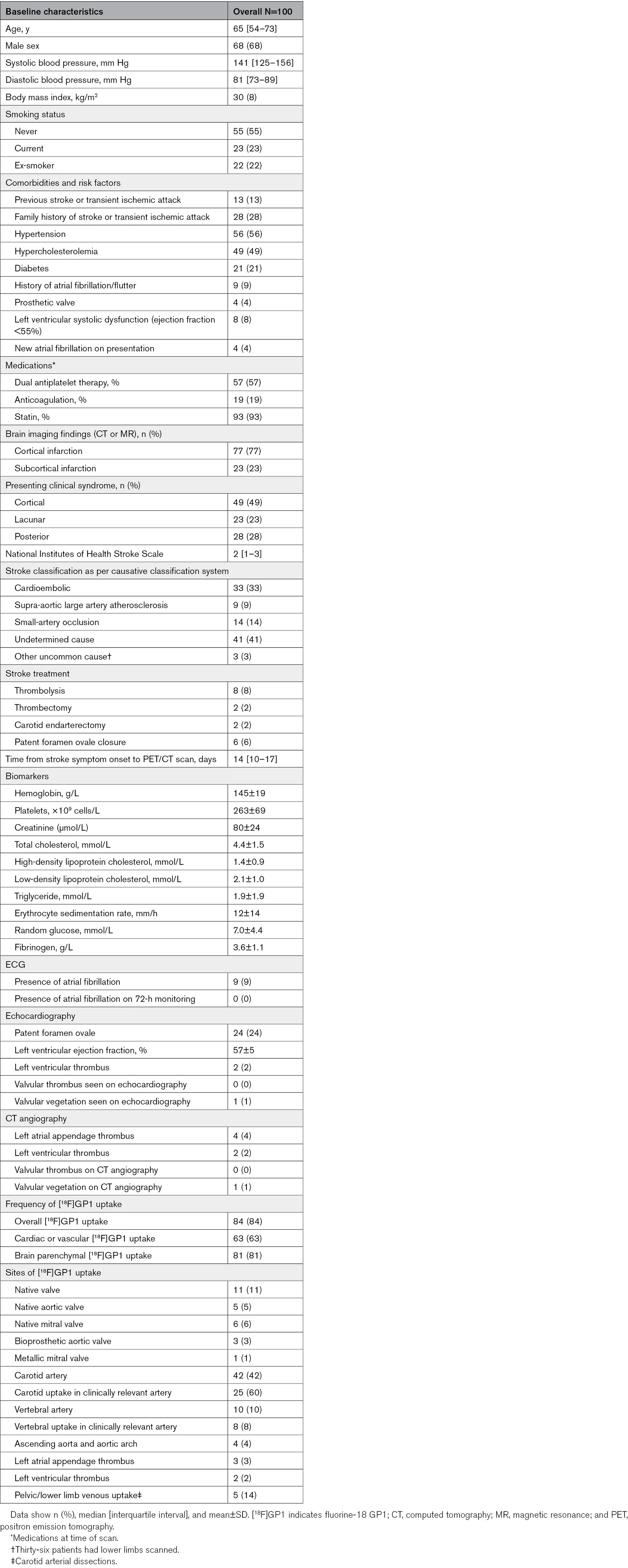
Baseline Characteristics of Study Participants

On brain CT or MRI, 77% of infarctions were cortical, and 23% subcortical. Median National Institutes of Health Stroke Scale score at first study visit was 2 (interquartile interval, 1–3), indicative of mild stroke severity. There was a high prevalence of stroke risk factors among recruited patients (Table 1). Four patients had a new presentation of atrial fibrillation, and 5 had a history of either permanent or paroxysmal atrial fibrillation. No new cases of atrial fibrillation were detected on prolonged (≥72 hours) ECG monitoring. A quarter of patients (n=24) had a patent foramen ovale on bubble-contrast echocardiography. Eight patients received thrombolysis, 2 mechanical thrombectomy, 2 carotid endarterectomy, and 6 patent foramen ovale closure.

### PET and CT Imaging

[^18^F]GP1 administrations and study-related procedures were well tolerated. No adverse drug reactions or serious adverse events were observed. Three adverse events did occur but were considered to be unrelated to [^18^F]GP1 by the investigator. All adverse events were mild and transient in nature. The average radiation burden for scans, excluding lower-limb imaging, was 11.3±2.1 mSv. The average radiation burden for total-body scans (including lower limbs) was 23.7±4.9 mSv. Overall, visual qualitative [^18^F]GP1 uptake was identified in 84 patients; 81 patients had brain parenchymal uptake, and 63 had cardiac or vascular uptake (Table 1). Regions of qualitative cardiovascular and brain parenchymal [^18^F]GP1 uptake (Figure S2) had higher quantitative uptake in the carotid arteries, left atrial appendage, cardiac valves, and brain parenchyma compared with regions with no [^18^F]GP1 uptake (Table S1; Figure S3). Both qualitative and quantitative assessments of [^18^F]GP1 uptake showed excellent intraobserver and interobserver agreement and discrimination (Table S2). There were no differences in the frequency or intensity of [^18^F]GP1 uptake according to the time to scan from symptom onset (Tables S3 and S4; Figure S4).

### Brain Uptake

Brain parenchymal [^18^F]GP1 uptake colocalized to areas of brain infarction identified on CT or MRI and was more commonly identified in patients who had cortical infarctions and strokes secondary to large artery atherosclerosis (Table S5). Brain parenchymal [^18^F]GP1 uptake was less likely to be seen in stroke secondary to small-artery occlusion (lacunar stroke) or in patients in whom cerebral infarctions were detected on MRI only (Table S5).

### Cardiovascular Uptake

#### Large-Artery Atherosclerotic Plaques

Forty-two patients had carotid [^18^F]GP1 uptake, with 25 (60%) being present within ipsilateral arteries supplying the territory of the index stroke (Figure 1; Figure S2). Eight patients had bilateral [^18^F]GP1 carotid artery uptake. In the remaining 17 (40%) patients, carotid [^18^F]GP1 uptake was on the side contralateral to cerebral infarction, with 2 cases of concomitant valvular [^18^F]GP1 uptake, 3 cases in the context of atrial fibrillation but no evidence of atrial appendage thrombus on [^18^F]GP1 PET/CT, and 1 case of a patent foramen ovale, but no lower-limb imaging was performed.

**Figure 1.**
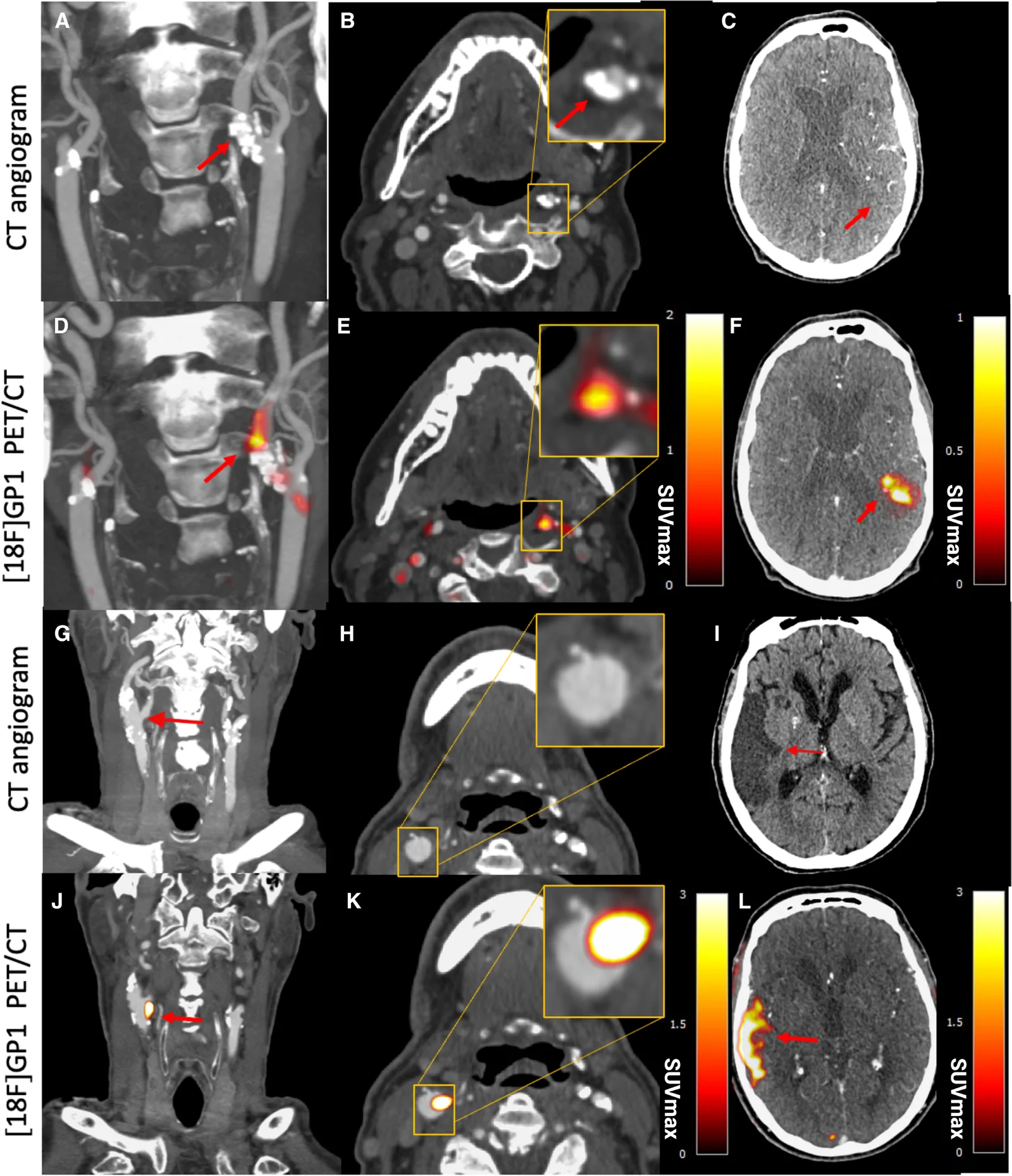
**Carotid artery and brain parenchymal fluorine-18 GP1 ([^18^F]GP1) uptake. A** through **F**, A case of an acute left temporal lobe infarction (red arrow, **C**) and bilateral mixed atherosclerotic plaque in carotid arteries with 60% to 65% stenosis of the left internal carotid artery (**A** and **B**). Stenosis was deemed to be below the surgical threshold (>70%), and the patient was not initially offered carotid endarterectomy. Despite bilateral carotid disease, [^18^F]GP1 uptake was only observed in the left internal carotid artery (**D** and **E**) and in the area of brain infarction (red arrow, **F**), suggestive of ipsilateral acute carotid plaque rupture and thromboembolism. The patient had a transient ischemic attack 10 days following his stroke in the same territory and underwent a carotid endarterectomy. **G** through **L** demonstrate a further case of an acute right middle cerebral infarction (red arrow, **I**). Again, despite bilateral mixed atherosclerotic plaque in the carotid arteries with 30% to 49% stenosis, [^18^F]GP1 uptake was only observed in the culprit right common carotid bifurcation (**J** and **K**), consistent with carotid artery thromboembolism and cerebral infarction. [^18^F]GP1 uptake was observed despite the degree of stenosis being below the surgical threshold for carotid endarterectomy. However, the carotid plaque demonstrates a high-risk phenotype with low attenuation features (red arrow, **G**). [^18^F]GP1 uptake was associated with the area of brain infarction (**I** and **L**). CT indicates computed tomography; PET, positron emission tomography; and SUV_max_, maximum standardized uptake value.

Carotid [^18^F]GP1 uptake was seen across all degrees of stenosis, although most carotid plaques with increased [^18^F]GP1 uptake demonstrated <50% stenosis (36 of 42, 86%). Carotid [^18^F]GP1 uptake correlated with increasing stenosis severity (Spearman ρ=0.37, *P*<0.001) (Table 2; Table S6), and the frequency of [^18^F]GP1 uptake within stenosis categories was higher in patients with ≥50% stenosis (6 of 8, 75%) compared with those with <50% (36 of 91, 40%) or no atheroma (3 of 31, 10%; all due to carotid dissection) (Figure S5; Table S6). Carotid [^18^F]GP1 uptake was associated with more mixed (58% versus 31%; odds ratio, 2.31 [95% CI, 1.05–5.07]; *P*=0.038), noncalcified (18% versus 9%; odds ratio, 5.56 [95% CI, 1.91–16.15]; *P*=0.002), and high-risk low-attenuation (38% versus 11%; odds ratio, 2.65 [95% CI, 1.29–5.44]; *P*=0.008) plaques on CT angiography (Table 2).

**Table 2.**
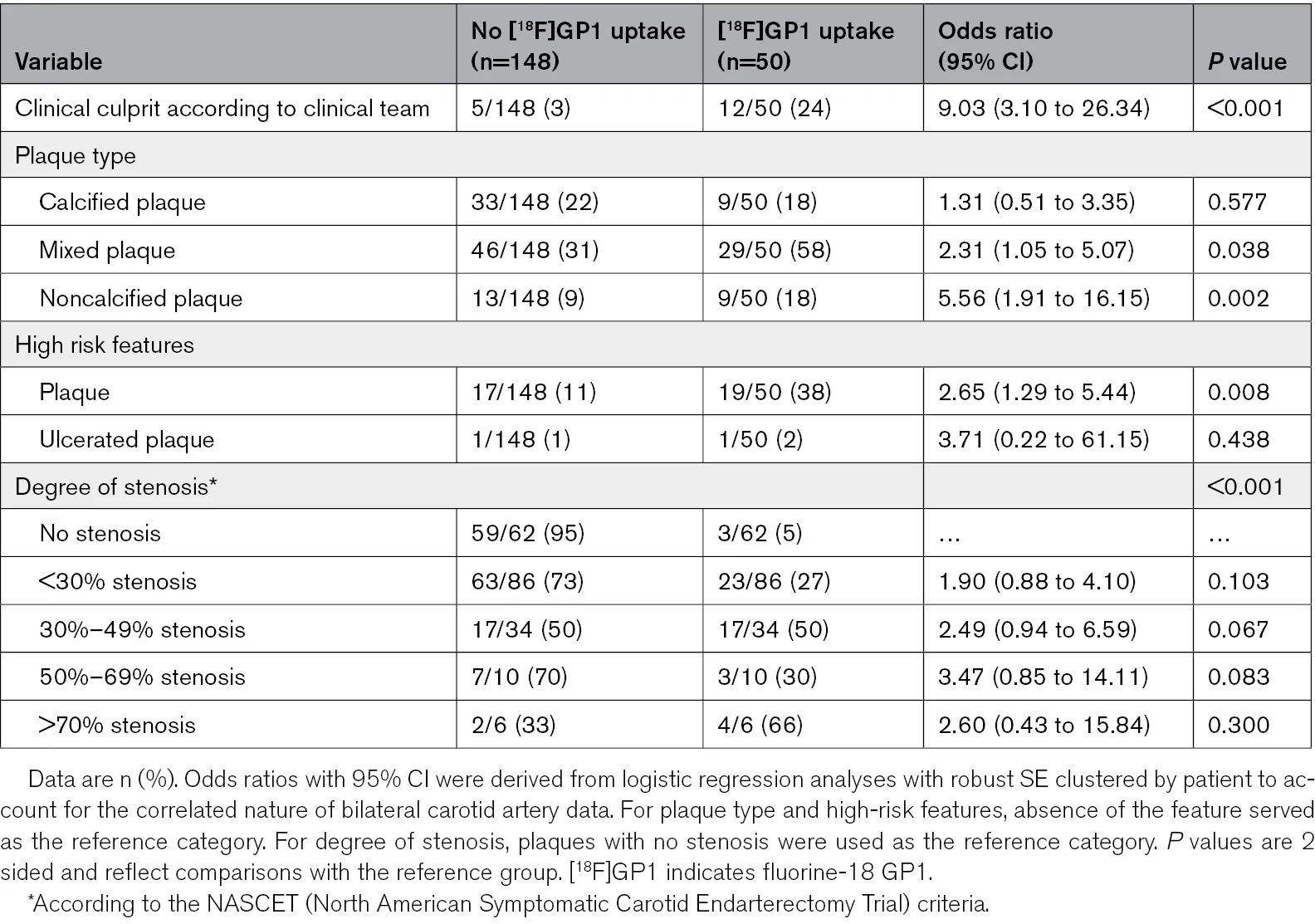
Association Between Carotid Plaque Characteristics and [^18^F]GP1 Uptake

#### Aortic and Mitral Valve

There were 11 cases of native cardiac valve [^18^F]GP1 uptake: 5 aortic and 6 mitral valves. One case was observed in the context of clinical and imaging evidence of native aortic valve endocarditis (Figure 2). Two cases of aortic valve [^18^F]GP1 uptake were observed in the context of calcific aortic stenosis: 1 patient with severe aortic stenosis and 1 with moderate stenosis of a bicuspid aortic valve (Figure 3; Figure S2). The 2 remaining cases of native aortic valve [^18^F]GP1 uptake were associated with noncalcific valve cusp thickening but no hemodynamically significant stenosis. Similarly, native mitral valve [^18^F]GP1 uptake was observed in the context of abnormal mitral valve anatomy, including leaflet thickening, calcification, or annular calcification (Figure 3). All 4 patients with prosthetic valves had valvular [^18^F]GP1 uptake without clinical, echocardiography or CT angiography evidence of valvular thrombosis or dysfunction.

**Figure 2.**
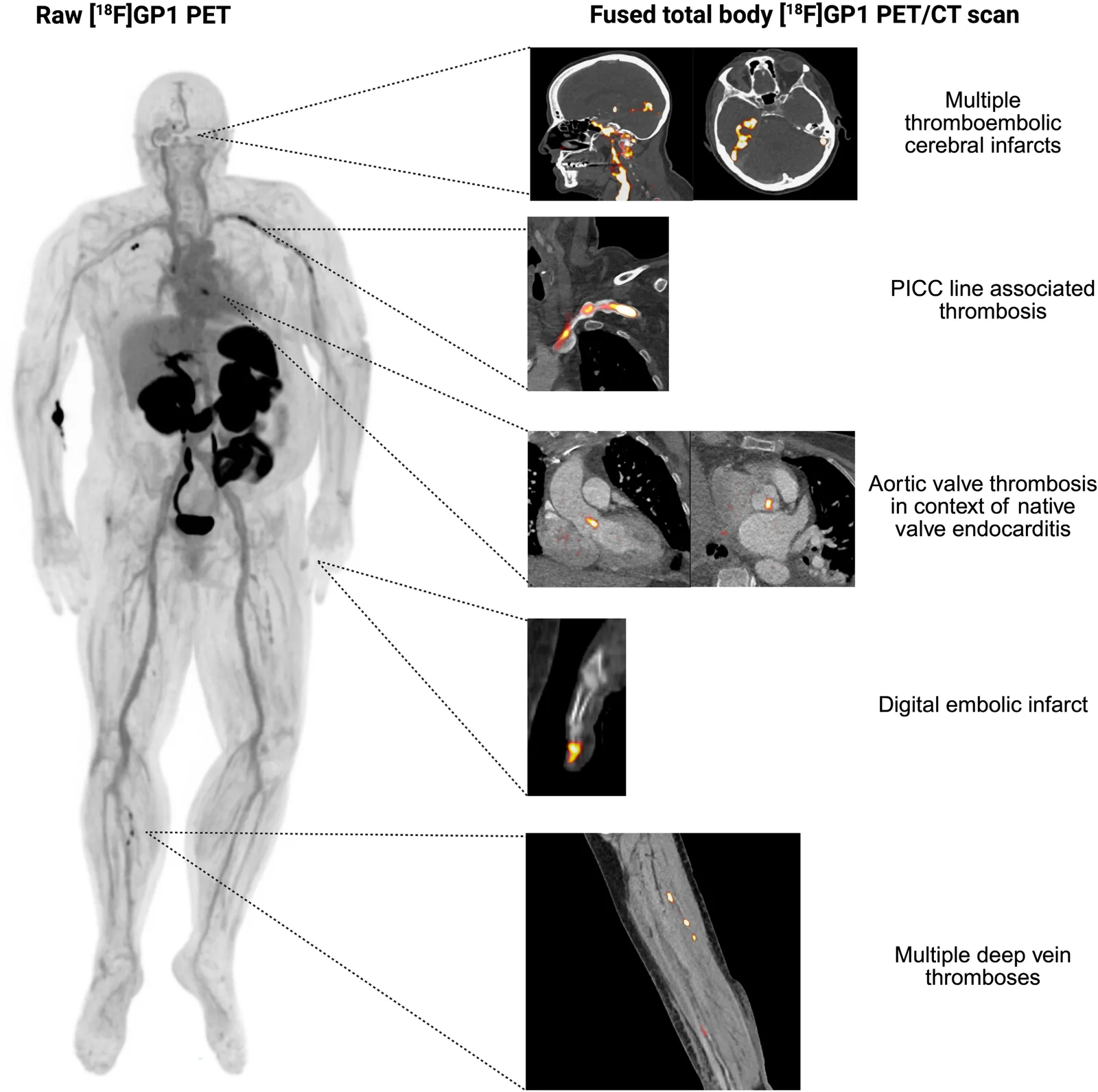
**Whole-body fluorine-18 GP1 ([^18^F]GP1) positron emission tomography (PET)/computed tomography (CT) imaging of systemic thrombosis secondary to aortic valve bacterial endocarditis.** Representative maximum-intensity projection raw PET images (**left**) and fused PET/CT images (**right**) demonstrate the ability of total-body [^18^F]GP1 PET/CT to detect active thrombus across multiple vascular territories within a single acquisition. Focal [^18^F]GP1 uptake consistent with platelet-rich thrombus is observed in the cerebral circulation (multiple thromboembolic infarcts), aortic valve (valve thrombosis in the context of clinically confirmed bacterial endocarditis), peripheral vasculature (digital embolic infarction), lower-limb deep veins (multiple deep vein thromboses), and around a peripherally inserted central catheter (PICC) (line-associated thrombosis). This figure illustrates the capacity of molecular imaging to provide a comprehensive, whole-body assessment of thromboembolic disease, including both native vessel and device-associated thrombosis, highlighting its potential to identify concurrent sources of thrombosis and to transform the investigation of thromboembolic disease from a region-specific to a systems-level approach.

**Figure 3.**
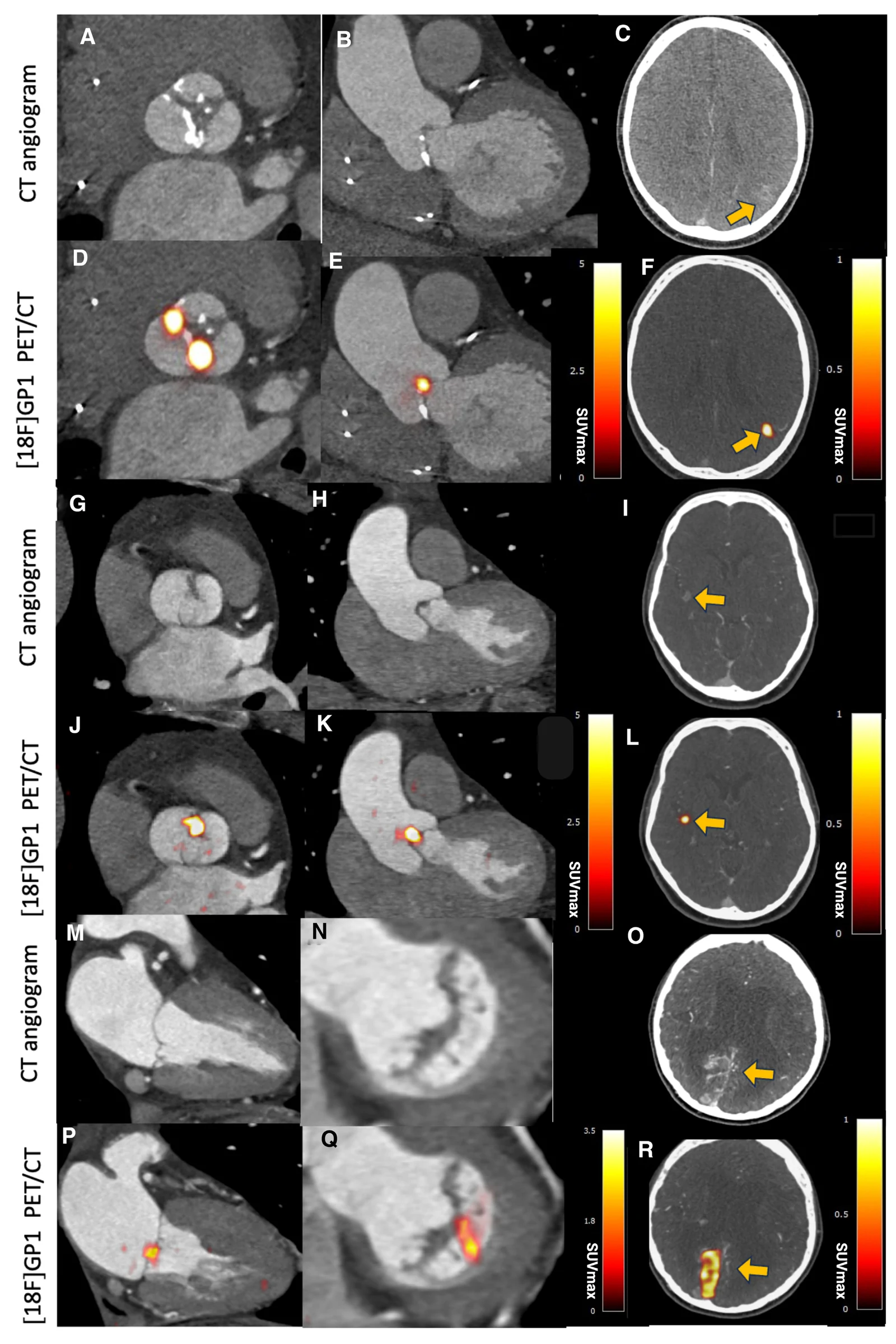
**Patients with ischemic stroke due to native cardiac valve disease.** Fluorine-18 GP1 ([^18^F]GP1) positron emission tomography (PET) and computed tomography (CT) angiography images of native valve uptake. **A** through **F**, a case of acute parietal lobe infarction (orange arrow, **C**) of undetermined etiology. Significant thickening and calcification of the aortic valve with severe stenosis (**A** and **B**) was seen on CT angiography. [^18^F]GP1 uptake was seen in the area of brain infarction (**F**) and noncoronary and right coronary cusps of the aortic valve (**D** and **E**), indicating cardioembolism as the cause of stroke. **G** through **L**, a case of a middle cerebral artery territory infarction (orange arrow, **I**) of undetermined etiology. **G** and **H**, a bicuspid aortic valve with cusp thickening and moderate aortic stenosis. **J** through **L**, [^18^F]GP1 uptake (orange red) in the area of brain infarction (**L**) and aortic valve (**J** and **K**). **M** through **R**, a case of an acute right posterior cerebral artery infarction (orange arrow, **O**) of undetermined etiology. **M** and **N**, Mild thickening of the native mitral valve on computed tomography. **P** through **R**, [^18^F]GP1 uptake (orange red) in the area of brain infarction (**R**) and posterior leaflet of the mitral valve (**P** and **Q**), suggesting cardioembolism as the cause of stroke. SUV_max_ indicates maximum standardized uptake value.

#### Left Atrial Appendage and Atrial Fibrillation

There were 9 patients who had a history of atrial fibrillation or newly identified atrial fibrillation on baseline ECG. Four cases of left atrial appendage thrombus were identified on CT imaging, with left atrial appendage [^18^F]GP1 uptake seen in 3 of these 4 cases (Figure S2; Figure S6).

#### Patent Foramen Ovale and Venous Thrombosis

CT venography was performed in a subset of 36 patients. Twenty-four patients had a patent foramen ovale identified on bubble-contrast echocardiography. Thirteen of these patients were over 60 years of age and were not considered candidates for a closure device according to current clinical guidelines. In 12 of these 13 patients, [^18^F]GP1 uptake was present in another clinically relevant artery or region, demonstrating an alternative underlying etiology (11 in the carotid arteries and 1 in the native aortic valve).

In the 11 patients who were younger than 60 years of age, 6 patients underwent percutaneous patent foramen ovale closure; 2 had confirmed lower-limb paradoxical emboli identified on [^18^F]GP1 PET/CT angiography, and a further 4 had no evidence of cardiovascular [^18^F]GP1 uptake, although they did not undergo lower-limb imaging. In the remaining 5 patients who did not undergo closure, the presence of a patent foramen ovale was thought clinically to be incidental due to either having lacunar infarcts on brain imaging (n=4) or already being on lifelong anticoagulation treatment (n=1).

There were 5 cases of pelvic or lower-limb venous [^18^F]GP1 uptake. One case was in the context of patent foramen ovale and a tibial fracture with an associated deep vein thrombosis (confirmed clinically on Doppler ultrasonography) (Figure 4). The other 4 cases demonstrated venous [^18^F]GP1 uptake in the lower limbs or pelvis, suggestive of subclinical deep vein thromboses. Two cases were in the context of patent foramen ovale and stroke of undetermined cause, with 1 patient having uptake within the patent foramen ovale itself (Figure 4), demonstrating thrombus in transit and confirming paradoxical embolization. The remaining 2 cases of subclinical deep vein thrombosis occurred in the context of sepsis from native aortic valve endocarditis or active cancer and in the absence of a patent foramen ovale on bubble-contrast echocardiography.

**Figure 4.**
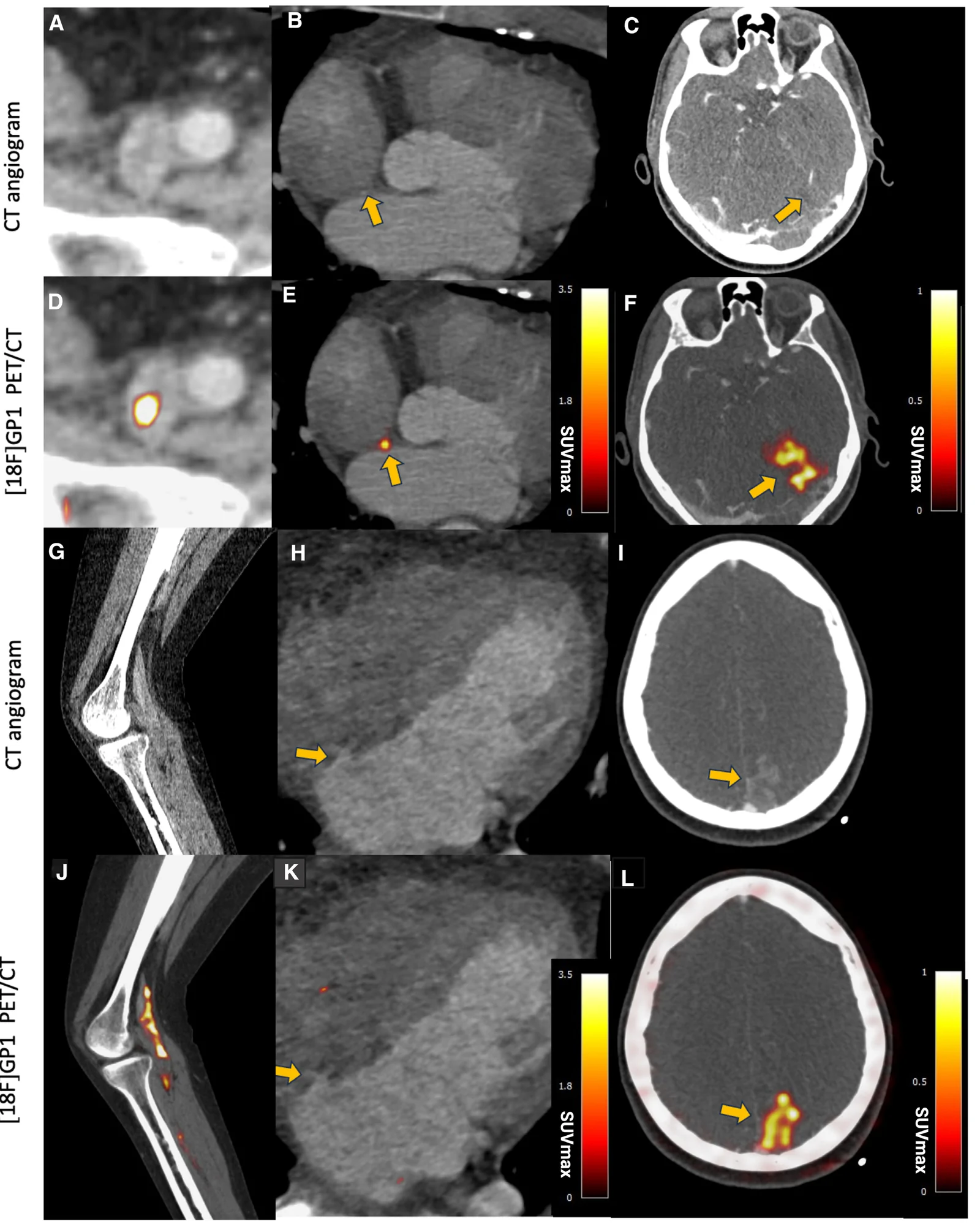
**Patients with ischemic stroke due to paradoxical embolism through a patent foramen ovale.** Shown are fluorine-18 GP1 ([^18^F]GP1) positron emission tomography (PET)/computed tomography (CT) images of subclinical deep vein thrombosis with paradoxical embolism through a patent foramen ovale, leading to posterior stroke. **A** through **F**, a case of an acute left cerebellar infarction (**C**, the orange arrow indicates subtle loss of gray matter differentiation). **A** and **B**, No clear obvious deep vein thrombosis on CT venography of the lower limbs (**A**) and a patent foramen ovale on CT of the heart (**B**). **D** through **F**, [^18^F]GP1 uptake (orange/red) in the area of brain infarction (**F**), right common femoral vein (**D**), and uptake over the patent foramen ovale (**E**). [^18^F]GP1 images are suggestive of a subclinical deep vein thrombosis with paradoxical embolism through a patent foramen ovale, leading to posterior stroke. **G** through **L**, a case of acute occipital stroke (**I**) in the context of a recent fibular fracture and deep vein thrombosis diagnosed on Doppler ultrasound. **G** and **H**, No clear deep vein thrombosis on the CT venogram of the lower limbs (**G**) and a patent foramen ovale on CT of the heart (**H**). **J** and **L**, [^18^F]GP1 uptake in the area of brain infarction (**L**) and left popliteal veins (**J**), indicating paradoxical embolism as the cause of stroke. SUV_max_ indicates maximum standardized uptake value.

### Reclassification of Stroke

Following declaration of the [^18^F]GP1 PET/CT findings, 26 patients had the etiology of their stroke changed, with 18 of 41 (44%) patients with stroke of undetermined being reclassified to either larger-artery atherosclerosis or cardioembolic causes. Within strokes classified as due to small-artery occlusion, 3 of 14 (21%) were reclassified to large-artery atherosclerotic disease or cardioembolism. Conversely, 4 of 33 (12%) of strokes deemed to be cardioembolic in nature were reclassified to large-artery atherosclerosis. No cases of large-artery atherosclerotic disease were reclassified (Figure 5).

**Figure 5.**
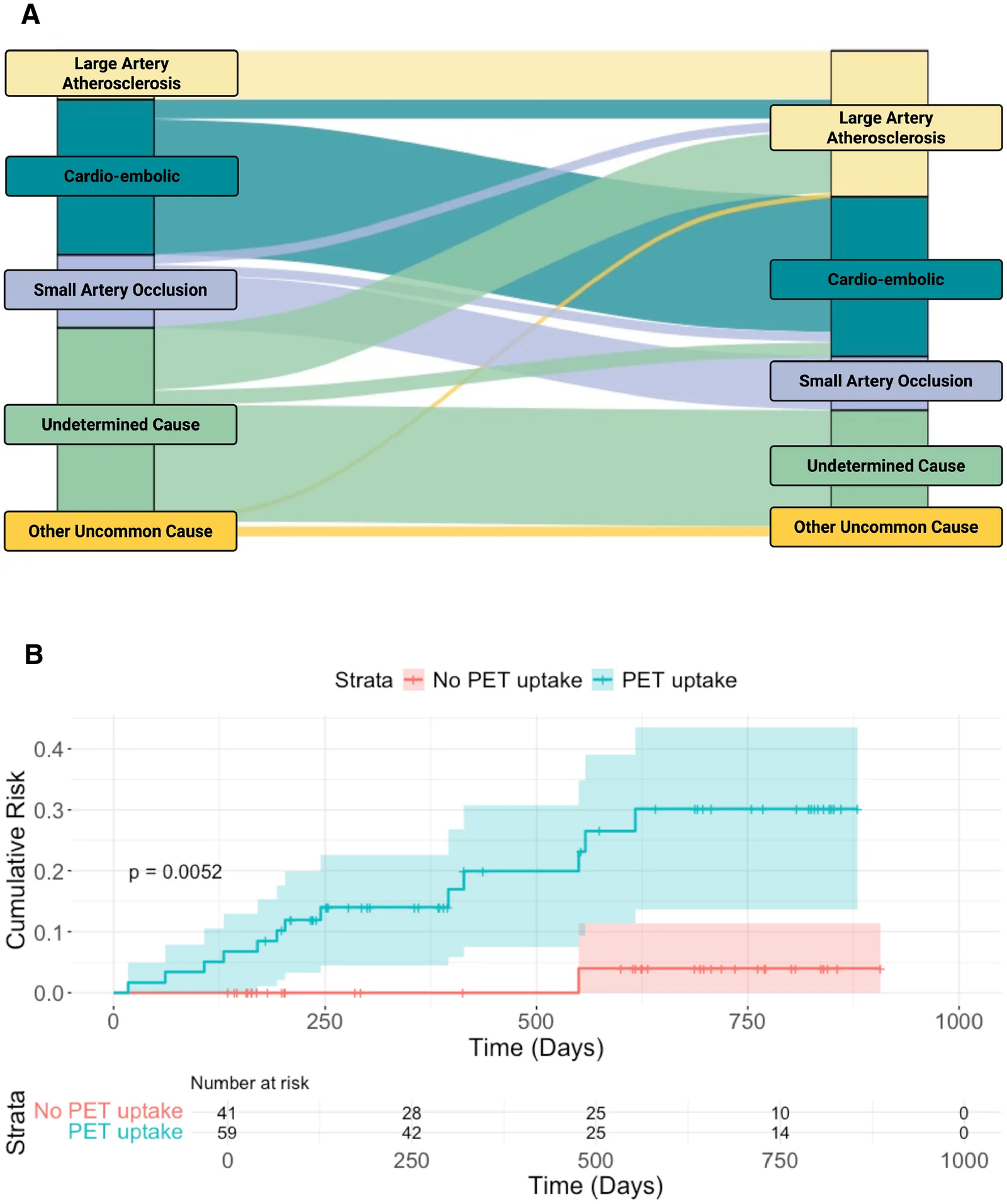
**Diagnostic reclassification and clinical outcomes. A**, Diagnostic reclassification expressed as an alluvial plot showing the percentage of patients with reclassification of stroke etiology following the results of the fluorine-18 GP1 positron emission tomography (PET) and computed tomography angiography imaging. **B**, Cumulative incidence plot of recurrent stroke or transient ischemic attack in patients with (turquoise) and without (red) cardiovascular fluorine-18 GP1uptake on positron emission tomography and computed tomography (log-rank *P*=0.0052).

### Ex Vivo Carotid Plaque Correlation

Ex vivo correlation of [^18^F]GP1 binding was performed in carotid atheroma specimens obtained from a separate group of 3 patients who had undergone in vivo [^18^F]GP1 PET/CT imaging before carotid endarterectomy, independent of the 100-patient cohort described above. In 2 cases, focal in vivo [^18^F]GP1 uptake corresponded to areas of intense ex vivo tracer binding on autoradiography and co-localized with histological evidence of platelet-rich thrombus on CD41 immunohistochemistry (Figure 6). Coincubation with tirofiban resulted in nearly complete inhibition of tracer binding (blocking 97% and 100%), confirming the specificity of [^18^F]GP1 for the glycoprotein IIb/IIIa receptor on activated platelets. In contrast, the third case demonstrated no focal in vivo [^18^F]GP1 uptake, no corresponding signal on autoradiography, and no CD41 staining on histological analysis.

**Figure 6.**
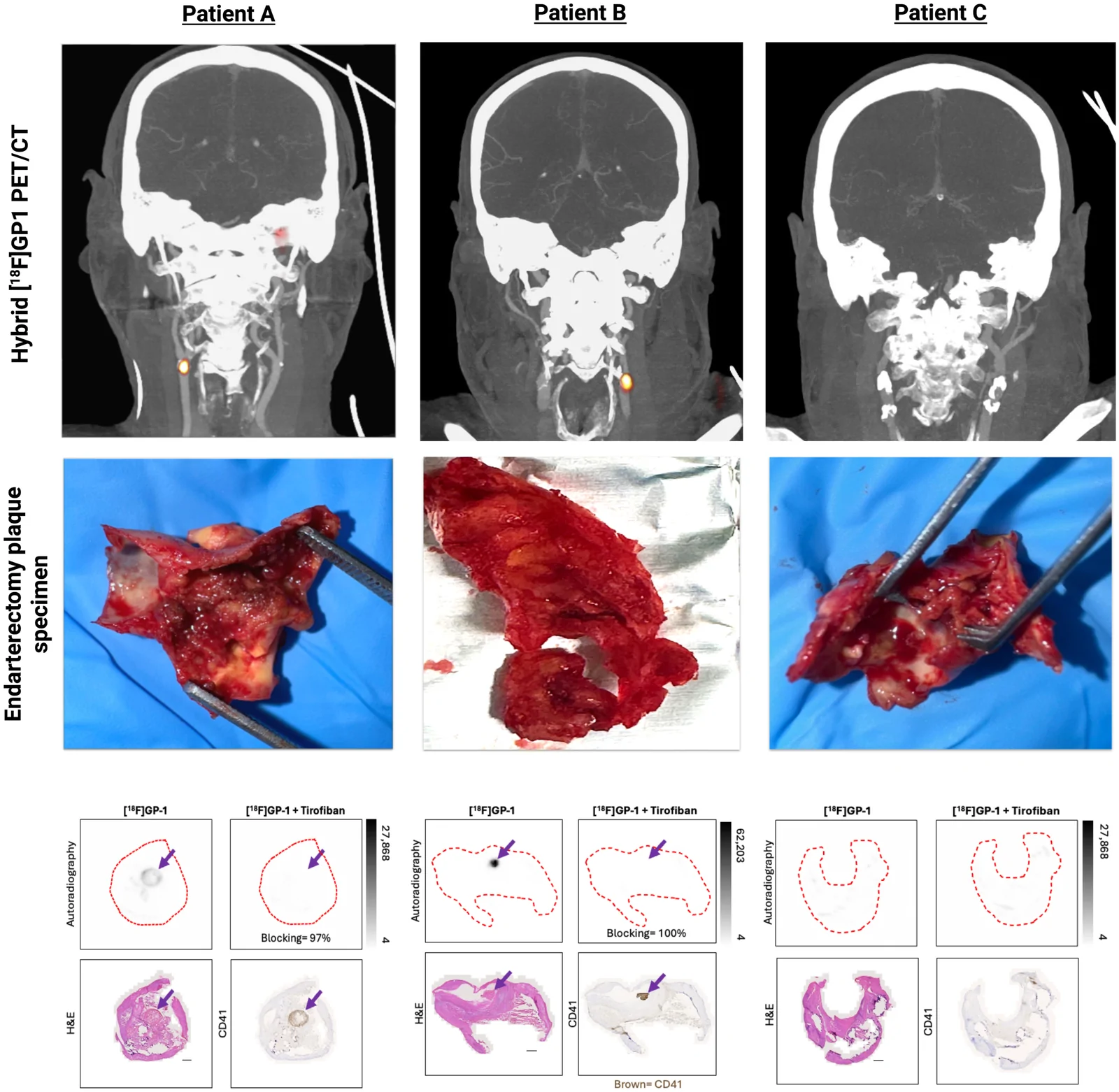
**Correlation of in vivo fluorine-18 GP1 ([^18^F]GP1) positron emission tomography (PET) and computed tomography (CT) angiography with ex vivo [^18^F]GP1 autoradiography and immunohistochemistry in patients with stroke undergoing carotid endarterectomy. Patient A** through **Patient C**, Representative examples of 2 patients with in vivo [^18^F]GP1 uptake (**Patient A** and **Patient B**) and 1 patient without uptake (**Patient C**), demonstrating concordance between in vivo [^18^F]GP1 positron emission tomography (PET)/computed tomography (CT) imaging and *ex vivo* tissue analysis. First row, Fused PET/CT images demonstrating focal [^18^F]GP1 uptake within carotid atheroma in 2 cases with no uptake in the third case. Second row, Corresponding surgically explanted carotid plaque specimens. Third row, Autoradiography demonstrating focal [^18^F]GP1 binding in the first 2 cases, with nearly complete inhibition following coincubation with tirofiban (a glycoprotein IIb/IIIa receptor antagonist) and absent uptake in the third patient. Bottom row, Histological assessment showing plaque morphology (hematoxylin and eosin [H&E]) and platelet-rich thrombus identified by CD41 immunohistochemistry (brown staining), corresponding to areas of tracer uptake. These findings demonstrate concordance between in vivo imaging, ex vivo tracer binding, and histological evidence of platelet accumulation, supporting the specificity of [^18^F]GP1 for activated platelets.

### Clinical Follow-Up

One hundred patients were followed up over a median duration of 613 (interquartile interval, 251–788) days with no deaths observed. Recurrent ischemic stroke or transient ischemic attack occurred in 14 patients, with 13 (93%) having cardiovascular [^18^F]GP1 uptake on their baseline PET/CT angiography imaging (log-rank *P*=0.005; Figure 5). In 12 of these 13 patients, [^18^F]GP1 uptake occurred in the relevant artery or other region implicated as the thromboembolic source of recurrent stroke or transient ischemic attack (ipsilateral carotid artery [n=7], vertebral artery [n=1], native mitral valve [n=1], and paradoxical embolism through patent foramen ovale [n=3]). Carotid stenosis was <50% in 6 (86%) of the 7 patients with ipsilateral carotid [^18^F]GP1 uptake.

Patients with cardiovascular [^18^F]GP1 uptake were 10.4-fold (95% CI, 1.35–79.40; *P*=0.024) more likely to experience a recurrent stroke or transient ischemic attack. Examination of Schoenfeld residuals indicated no violation of the proportional hazard assumption for the Cox regression model (*P*=0.48; global test *P*>0.050). No other traditional cardiovascular risk factors were associated with recurrence, including hypertension, smoking status, diabetes, hypercholesterolemia, carotid stenosis ≥50%, and prior history of stroke or transient ischemic attack. In an exploratory multivariable model, cardiovascular [^18^F]GP1 uptake remained independently associated with an increased risk of recurrent stroke or transient ischemic attack after adjusting for traditional vascular risk factors (Table S7).

Among patients with recurrent events, treatment at baseline was heterogeneous and not guided by [^18^F]GP1 PET/CT findings. For carotid and vertebral artery uptake, dual antiplatelet therapy was given for 21 days, followed by single antiplatelet therapy according to current guidelines. Among those with paradoxical embolism, 1 patient received lifelong anticoagulation treatment, and 2 received anticoagulation treatment before undergoing patent foramen ovale closure in accordance with standard clinical practice.

## Discussion

We investigated the use of [^18^F]GP1 PET/CT angiography for the noninvasive detection of thrombosis and thromboembolism in patients with acute ischemic stroke. The presence of activated platelets using [^18^F]GP1 was detected within the brain parenchyma and cardiovascular system in a high proportion of patients across all ischemic stroke etiologies, and this has several intriguing and clinically important implications. First, [^18^F]GP1 PET/CT identified a source of potential thromboembolism in a large proportion of patients with stroke of undetermined etiology, resulting in many being reclassified. Second, we identified 2 important and relatively frequent causes of stroke that warrant greater recognition: native cardiac valve thrombosis and ipsilateral carotid atherothrombosis in plaques with <50% stenosis. Third, we found that thrombosis in atrial fibrillation and patent foramen ovale is less frequent than commonly presumed. Finally, the presence of cardiovascular [^18^F]GP1 uptake appears to be a major determinant of recurrent stroke or transient ischemic attack, underlining the pathogenic importance of thromboembolism and the need for enhanced stroke diagnosis and more focused preventative strategies.

Our work represents an important shift from inference-based to mechanism-based stroke classification. Currently, there are no investigations that can specifically identify the underlying mechanism of a patient’s stroke. Patients are screened for conditions that are associated with the risk of stroke, such as carotid stenosis ≥50% or atrial fibrillation, and it is assumed that these have caused the incident stroke; ie, inference-based diagnosis and clinical management. Our data with [^18^F]GP1 PET indicate that the current inference-based approach may be incorrect or fails to identify the cause of the stroke in a substantial proportion of cases. Indeed, this mechanism-based approach of [^18^F]GP1 PET has identified previously underappreciated causes of stroke, questioned the magnitude of the contribution of several risk factors, and may provide a framework to target pharmacological therapies or interventions to those most likely to benefit and prevent stroke recurrence.

The commonest sites for cardiovascular [^18^F]GP1 uptake were the carotid arteries. The likelihood of carotid [^18^F]GP1 uptake progressively increased with stenosis severity so that 75% of carotid plaques with stenosis >50% had [^18^F]GP1 uptake, which is consistent with a prior histological study.^19^ However, within our cohort, approximately 80% of carotid plaques with [^18^F]GP1 uptake had a stenosis of <50%. This is analogous to coronary artery disease, where 80% of myocardial infarctions occur on nonobstructive coronary plaques, although the likelihood of myocardial infarction increases with stenosis severity. Furthermore, [^18^F]GP1 uptake was observed more frequently in mixed calcified and noncalcified plaques and plaques with high-risk features on CT angiography. This is consistent with previous findings that such plaque types are more vulnerable, pathologically active, and prone to atherothrombotic events compared with more stable calcified plaque.^20,21^ Furthermore, the presence of ipsilateral [^18^F]GP1-positive thrombosis in those with <50% stenosis appears to be a major determinant of recurrent stroke or transient ischemic attack.

In an individual patient data meta-analysis of the seminal randomized controlled trials,^22^ patients with ≥50% stenosis gained most clinical benefit from carotid endarterectomy surgery. Below 50% stenosis, any benefit from carotid endarterectomy was offset by the hazard of periprocedural stroke from the surgery itself. Our findings may explain the apparent balance of risk and benefit in these patient groups. For patients with stenosis ≥50%, the majority will have a ruptured or complex plaque with in situ thrombosis, and the risk of recurrent events will be high, meaning the benefits of carotid endarterectomy will outweigh the hazard of procedural stroke. Conversely, patients with stenosis <50% will have a lower frequency of plaque rupture and in situ thrombosis, meaning that the risk of procedural stroke nullifies or exceeds the reduction in recurrent stroke. This does beg the question of whether a thrombus-based approach may be superior to the current stenosis-based approach, especially since most recurrent events occurred in patients with carotid stenosis <50%. For example, selection of patients with plaque thrombosis may identify those with carotid stenosis <50% who would benefit from intervention due to unstable or ruptured plaque. Conversely, this approach could also avoid unnecessary surgery in those with stenosis ≥50% and no in situ thrombus, thereby further enhancing the known overall benefits in this patient group. However, this hypothesis requires further evaluation in future studies.

Valvular heart disease is a known risk factor for cardioembolic stroke, but at present, only prosthetic valves, valve endocarditis, and mitral stenosis are included in current stroke etiology classification systems.^23^ We identified [^18^F]GP1 uptake on native aortic and mitral valves. Native valve thrombus formation was thought to be a rare phenomenon and has most often been reported in the context of infectious or marantic endocarditis.^24,25^ Where we identified valvular [^18^F]GP1 uptake, this was associated with a morphologically abnormal valve with leaflet thickening or calcification, which included 2 cases of valvular [^18^F]GP1 uptake in more advanced moderate or severe aortic stenosis. Our study is the first to demonstrate that native valve thrombosis occurs in a substantial proportion of patients with ischemic stroke and perhaps represents a more frequent and previously unidentified source of thromboembolism. These preliminary findings require further investigation and validation in larger cohorts, with clinical consideration of whether such patients would benefit from additional anticoagulation therapies or valve intervention. Indeed, our findings may explain the recent paradoxical finding that early aortic valve intervention in patients with asymptomatic aortic stenosis not only does not increase stroke rates but appears to reduce them.^26–28^

Atrial fibrillation is a widely recognized risk factor for ischemic stroke, which is due to thromboembolism from the left atrial appendage. It is often assumed that it is the predominant cause of stroke in patients with atrial fibrillation. Our study suggests that this may be misplaced, since left atrial appendage thrombus was only identified in the minority of patients with atrial fibrillation. We acknowledge that [^18^F]GP1 PET imaging cannot definitively rule out the previous presence of left atrial appendage thrombus, which may have completely embolized or resolved with antithrombotic therapy by the time of scanning. However, as we have demonstrated, patients often have multiple competing causes, such as carotid atheroma, which may perhaps be overlooked or underinvestigated when the cause of stroke is inferred from presumed left atrial appendage thrombus from atrial fibrillation. Although some trials have suggested a reduction in stroke,^29^ our findings are consistent with the recent STABLED (Stroke Secondary Prevention with Catheter Ablation and Edoxaban for Patients with Nonvalvular Atrial Fibrillation) trial, where ablation of atrial fibrillation had no demonstrable effect on recurrent stroke events.^30^

It can be difficult to ascertain whether a patent foramen ovale is causatively associated with ischemic stroke. The observed prevalence of patent foramen ovale in our study is similar to that described previously within the general population.^31^ In patients with a clinical suspicion of underlying paradoxical embolization through a patent foramen ovale (below 60 years of age, stroke of undetermined cause, no other potential sources identified), clinical and subclinical deep vein thromboses were identified within the pelvis and lower limbs using [^18^F]GP1 PET/CT imaging. Perhaps more importantly, [^18^F]GP1 PET/CT angiography identified an alternative underlying etiology of stroke in most patients with a patent foramen ovale who were over 60 years of age. This is meaningful, as decisions regarding closure of a patent foramen ovale remain challenging in current clinical practice due to confounding co-existing risk factors, especially in patients over the age of 60 years or when other comorbidities are observed in patients younger than 60 years (such as mild <50% carotid stenosis). There are currently ongoing clinical trials (NCT05907694 and NCT0538794) exploring patent foramen ovale closure in patients over 60 years who will inherently have several other potential causes for stroke. We suggest that [^18^F]GP1 PET/CT angiography may aid in selecting those who may truly benefit from a closure device by excluding other, more common causes of ischemic stroke and identifying venous thromboses with or without thrombus in transit within the foramen ovale.

Brain parenchymal [^18^F]GP1 uptake was seen in most patients, and this corresponded to areas of infarction on CT or MRI. Our previous ex vivo postmortem brain tissue study showed that this represents microvascular obstruction and petechial hemorrhage within the brain parenchyma.^7^ Cerebral infarctions in which there was no observed [^18^F]GP1 uptake were small and predominantly subcortical infarctions and less likely to be due to embolic phenomena. Indeed, we demonstrated that only a small proportion of lacunar infarctions (11%) were due an embolic source, and the majority appear to be due local mechanisms, in keeping with our current understanding of nonthrombotic small-vessel disease.^32^

The concept of radiolabeled thrombus imaging has a long history. Early single-photon emission CT approaches, including indium 111–labeled platelets, iodine 131–labeled fibrin-targeted monoclonal antibodies, and technetium 99m–labeled glycoprotein IIb/IIIa antagonists demonstrated the proof of concept for in vivo thrombus detection.^33–35^ However, these approaches were limited by lower spatial resolution, longer acquisition times, and unfavorable radiation dosimetry compared with modern PET tracers, and none were evaluated specifically within an ischemic stroke population. Beyond these historical approaches, other molecular targets have been proposed for thrombus imaging. Positron-emitting fibrin-binding probes have been evaluated predominantly in preclinical models, with limited human data to date.^36–38^ In a small case-control study, Izquierdo-Garcia et al demonstrated the feasibility of detecting left atrial appendage thrombus using the fibrin-targeted tracer [^64^Cu]FBP8 in patients with atrial fibrillation. However, fibrin-targeted PET imaging has not yet been evaluated for detecting atherothrombosis or thromboembolism in stroke. Although both activated platelets and fibrin are present in all thrombi, an arterial thrombus typically forms under high-shear conditions with a predominance of platelets, whereas a venous thrombus occurs in low-shear environments and is more fibrin rich. Platelet-targeted probes may therefore be more sensitive for arterial thrombi. Comparative studies are required to identify the best ligand and approach to identify stroke-associated thromboembolism.

There are several limitations of our study. Our study population did not include patients with stroke and severe functional impairment, given their inability to tolerate the imaging protocol. Our cohort had a relatively low rate of reperfusion therapy, reflecting the mild stroke severity and the inclusion of patients presenting outside the thrombolysis treatment window. The time between symptom onset and [^18^F]GP1 PET/CT angiography imaging was arguably too long, given that thrombus formation and dissolution is a dynamic process and is affected not only by time but also the use of antithrombotic therapies. However, it was reassuring that there was no difference in time to scan between those with or without [^18^F]GP1 uptake. These findings suggest that thrombus associated with thromboembolic stroke takes time to resolve and that there are ongoing stimuli for platelet adhesion and thrombus formation from diseased vessels, valves, and tissues. Clinically, this is consistent with the highest risk of recurrent stroke being in the first 30 days after an index stroke. We acknowledge the need for longitudinal data with repeated [^18^F]GP1 PET/CT imaging to explore changes in uptake with time and the effect of therapeutic interventions. The use of 2 PET/CT platforms is a further study limitation, although we observed no major differences in qualitative or quantitative measures of [^18^F]GP1 uptake rates between scanners, suggesting that our findings are likely to be reproducible across PET/CT platforms. Notwithstanding these considerations, further studies with larger sample sizes and multiple centers are needed to demonstrate generalizability and to establish the clinical efficacy and usefulness of this technique. Finally, we recognize that the performance of multiple subgroup and sensitivity analyses introduces risk of type 1 error, although these analyses were conducted in an exploratory and hypotheses-generating manner.

In conclusion, hybrid [^18^F]GP1 PET/CT angiography in patients with acute ischemic stroke identified plausible sources of thrombosis across all stroke etiologies and resulted in stroke reclassification in a large proportion of cases, especially in those with an undetermined etiology. Importantly, it has highlighted the important role of carotid arteries with <50% stenosis and native valve thrombosis as previously unidentified sources of thromboembolism. Conversely, atrial fibrillation and a patent foramen ovale may be incidental and may not be the causal mechanism of stroke. [^18^F]GP1 PET holds major promise for improving our understanding of the role and origin of thrombosis in ischemic stroke, shifting the paradigm from inference-based to mechanism-based stroke classification, thereby holding major potential to guide clinical diagnosis and pharmacological and procedural management and improve clinical outcomes in patients with ischemic stroke.

## Article Information

### Sources of Funding

Dr Whittington (FS/CRTF/21/24129), Dr Dweck (FS/SCRF/21/32010), Dr Williams (FS/ICRF/20/26002), and Dr Newby (CH/09/002/26360, RG/F/22/110093, RE/24/130012, and SI/F/26/21170025) are supported by the British Heart Foundation. Dr Balmforth (MR/Y009770/1) is supported by the Medical Research Council. The Scottish Total-body PET Facility is supported by the Medical Research Council as part of the National PET Imaging Platform (MR/Y008944/1). Edinburgh Imaging and the Edinburgh Clinical Research Facility are supported by the NHS Lothian Health Board through NHS Research Scotland and the Chief Scientist Office of Scottish Government.

### Disclosures

None.

### Supplemental Material

Supplemental Methods

Supplemental Data

Figures S1–S6

Tables S1–S7

## Supplementary Material
