# Supplementary material for: Noninvasive Thrombus Imaging in Patients With Ischemic Stroke
Source: Circulation. 2026 Sep 1;154(15):1424–40. doi: 10.1161/CIRCULATIONAHA.126.080420 (PMC13668038; doi:10.1161/CIRCULATIONAHA.126.080420)
Supplement: Supplementary file 1 [file cir-154-1424-s001.pdf]

Supplementary Material

Supplementary Methods

[^{18}F]GP1 Radiosynthesis

Radiosynthesis of [^{18}F]GP1 was carried out using a NEPTIS Perform synthesizer (Optimized Radiochemical Applications, Neuville, Belgium). The synthesis was a two-step process, involving the nucleophilic fluorination of the tosylate precursor (GP1 precursor) followed by the removal of the protecting groups [12]. After neutralization and separation by semi-preparative high-performance liquid chromatography, [^{18}F]GP1 was then formulated in phosphate buffer before being terminally sterilized in an autoclave.

[^{18}F]GP1 Positron Emission Tomography and Computed Tomography Angiography

All patients underwent [^{18}F]GP1 PET/CT on one of two hybrid scanners with prospective electrocardiogram-gating: a 64-multislice PET/CT scanner (Biograph mCT, Siemens, Germany, 70% of participants) or a long-axial field of view 128-multislice PET/CT scanner (Siemens Biograph Vision Quadra; software version VR20C, 30 of participants) which differ in sensitivity and spatial resolution. Acquisition and reconstruction parameters were selected and optimized for each system to ensure clinically appropriate image quality and quantitative performance, rather than harmonizing reconstruction settings across platforms. Quantitative standardized uptake values (SUV) harmonization was not applied, as the primary endpoint relied on qualitative assessment and relative uptake, rather than absolute SUV quantification. Uptake was defined primarily using a target-to-background ratio (TBR) which mitigates inter-scanner variability by referencing activity to blood pool.

Positron emission tomography list mode acquisition was performed 60-min after intravenous injection of a target dose of 250 MBq [^{18}F]GP1 with 20-min bed positions centered over the aortic arch, carotid arteries and brain, or continuous bed motion with a nominal scan speed of 1.5 mm/sec, resulting in scan times of approximately 20 min for a vertex-to-toes anatomical range. For qualitative visual assessment of [^{18}F]GP1, reconstruction was performed using ordered subset expectation maximization with point-spread-function and time-of-flight capabilities enabled undergoing 4 iterations of 5 subsets. Matrix size was 440x440 giving pixel spacing of 1·65x1·65 mm^2, slice thickness of 3 mm and Gaussian smoothing of 3 mm. This was acquired in 'ultra-high sensitivity' mode and was attenuation corrected. For quantitative analysis, reconstruction was performed using ordered subset expectation maximization with time-of-flight capabilities enabled undergoing 4 iterations of 5 subsets. Matrix size was 440x440 giving pixel spacing of 1·65x1·65 mm^2, slice thickness 3 mm, Gaussian smoothing of 3 mm. Acquired in 'high sensitivity' mode and was attenuation corrected.

Immediately after PET acquisition and CT attenuation correction, contrast-enhanced CT angiography of the heart and major thoracic arteries was performed using 70 mL of iodinated contrast medium with a flow of 5-6 mL/s after determining an appropriate trigger determined by a test bolus of 15 mL contrast material. CT angiography was ECG-gated. The cerebral and carotid circulation was then assessed using 50 mL of iodinated contrast medium. Helical CT was performed with tube voltage and tube current selected automatically based on scout images. CT venography of the abdomen, pelvis and lower limbs was performed to assess for venous thrombosis. This was undertaken by delayed scanning 25 seconds after the injection of iodinated contrast medium for the thoracic CT angiography.

Carotid and Vertebral Artery [^{18}F]GP1 Image Analysis

Assessment of [^{18}F]GP1 uptake was performed on both carotid arteries and vertebral arteries. For an artery to be classed as positive for [^{18}F]GP1, uptake had to be associated with (i) atherosclerotic plaque, seen in three contiguous axial CT slices in three different orthogonal planes, (ii) follow the vessel contour and (iii) be above the background blood pool, with a quantitative confirmation defined a priori as $TBR_{max} \geq 1.25$ as described previously [34]. For quantitative analysis, 3-dimensional spherical volumes were placed over the area of maximum signal with the diameter of volume of interest adjusted to match the size of vessel. Regions of interest were defined on co-registered PET and CT images and were carefully positioned to correspond to the vascular lumen and adjacent plaque where applicable. These were delineated using anatomical guidance from CT angiography to ensure consistent placement. The internal jugular vein at the level of the carotid bifurcation was used as the background blood pool activity and an average of three maximum standardized uptake values was taken as described previously [15]. The mean and maximum standardized uptake values were measured and corrected for blood pool activity which was used to generate a target-to-background ratio. In the absence of carotid artery [^{18}F]GP1 uptake, measurements were taken at the largest atheromatous plaque within the carotid bifurcation or in the proximal 1 cm of the internal carotid artery if no plaque was present as described previously [35, 36].

Valvular [^{18}F]GP1 Image Analysis

Both the aortic and mitral valves were assessed for [^{18}F]GP1 uptake using a standardized method [37, 38]. Following co-localization of the diastolic PET and CT images, the aortic valve was evaluated in three longitudinal views and an *en-face* view, and the mitral valve was assessed in three longitudinal views. For both valves, the PET signal windowing was adjusted to enable visual determination of the maximum valvular uptake. This qualitative assessment determined whether the valve had [^{18}F]GP1 uptake, with a quantitative confirmation defined a

priori as $TBR_{max} \geq 2.0$ (to account for higher intracardiac background activity). For quantitative assessment of the aortic valve, a volume of interest was created by a 3-dimensional polyhedron which was contoured to the valve annulus with a height of 6-mm similar to previous studies [39, 40]. For the mitral valve, a volume of interest was created using a 10-mm sphere which was centered over the maximum voxel of activity in valves or the commissures of the valve in those without uptake. The SUV_{max} values were measured and corrected for blood pool activity which was measured in a cylinder of 8-mm radius and 9-mm height in the right atrium to generate a TBR_{max}.

Brain [^{18}F]GP1 Image Analysis

Visual and quantitative assessment of brain uptake was performed as described previously [41, 42]. [^{18}F]GP1 uptake was determined using 3-dimensional spherical volumes of interest centered on the region of interest adjusted to encompass the area of uptake to give maximum SUV. If there was no visual [^{18}F]GP1 uptake, the volume of interest included the infarct area as seen on CT or MRI. To determine the brain background activity, 3-dimensional spherical volumes were placed over the contralateral non-infarcted brain tissue and an average of maximum standardized uptake values was taken as described previously [41, 42].

Abdominal, Pelvic and Lower Limb Venous [^{18}F]GP1 Image Analysis

Co-registration of the CT venogram and PET images was performed using fixed structures, such the bones of the pelvis and femurs. [^{18}F]GP1 uptake within the venous system was determined by windowing the PET images to a level where background blood pool was removed. Quantitative uptake was determined by 3-dimensional spherical volumes encompassing the region of greatest uptake. To measure blood pool activity, an average of

three SUV_{mean} measurements from 3-dimensional spherical volumes in infrarenal inferior vena cava were used.

Intra- and inter-reader agreement for [^{18}F]GP1 qualitative and quantitative PET uptake

Analysis of qualitative and quantitative [^{18}F]GP1 PET uptake (TBR_{max}, SUV_{max}, TBR_{mean} and SUV_{mean}) intra- and inter-observer repeatability were assessed in the carotid arteries (n=198) and native cardiac valves (n=40) using descriptive statistics with intraclass correlation coefficient and Bland-Altman with mean bias and 95% limits of agreement by 2 independent expert readers who were blinded to all clinical data and CCS classification. Based on 95% confidence intervals of the intraclass correlation coefficient values, <0.5, 0.5-0.75, 0.75-0.9 and >0.90 indicate poor, moderate, good and excellent repeatability respectively.

Exploratory Receiver Operating Characteristic analyses

For future studies, a fully objective threshold-based classification with blinded application represents an ideal analytical framework. In the present study, quantitative thresholds were prespecified and applied in a blinded manner to validate visual interpretation rather than to serve as the primary classifier, reflecting the proof-of-concept nature of the work and the biological heterogeneity of platelet signal across vascular territories. Exploratory Receiver Operating Characteristic analyses were also performed to assess data-driven thresholds using Youden's index across different vascular territories.

Supplementary Data

Intra- and inter-reader agreement for [^{18}F]GP1 qualitative and quantitative PET uptake

Agreement for qualitative identification of [^{18}F]GP1 uptake in the carotid arteries was excellent. Intra-reader agreement was near perfect (Cohen's κ = 0.99), with an overall agreement of 98.0%. For native cardiac valve uptake (aortic and mitral), which has not been previously described, qualitative agreement was perfect in a subset of 40 participants (100% intra- and inter-reader agreement).

For carotid quantitative reproducibility, intra-observer intraclass correlation coefficients (ICC) ranged from 0.851–0.916 across SUV and TBR metrics, and inter-observer ICCs ranged from 0.810–0.899. Bland–Altman analyses demonstrated minimal systematic bias with narrow limits of agreement, indicating good repeatability and reproducibility across all carotid uptake parameters. Similarly, for native cardiac valvular measurements, reproducibility was excellent, with intra-observer ICCs ranging from 0.98–1.00 and inter-observer ICCs ranging from 0.87–0.98 across SUV and TBR metrics, again with minimal bias on Bland–Altman analysis (***Supplementary Table 2***).

Exploratory Receiver Operating Characteristic analyses

For vascular (carotid) imaging, a predefined threshold of $TBR_{max} \geq 1.25$ relative to blood pool, consistent with prior literature [6], was applied. This demonstrated excellent concordance with blinded expert visual reads, with sensitivity 96%, specificity 91%, positive predictive value (PPV) 80%, and negative predictive value (NPV) 99%. For valvular imaging, a predefined threshold of $TBR_{max} \geq 2.0$ was used to account for higher intracardiac background activity, yielding sensitivity 100%, specificity 96%, PPV 70%, and NPV 100%.

Exploratory Receiver Operating Characteristic analyses were performed to assess data-driven thresholds using Youden's index. For carotid imaging, the Youden-derived threshold was TBR_{max} 1.285, yielding sensitivity 94%, specificity 98%, PPV 94%, and NPV 98%, closely aligning with the prespecified threshold of 1.25. For cardiac imaging, the optimal TBR_{max} threshold was 2.09, yielding sensitivity 100%, specificity 97%, PPV 76%, and NPV 100%; performance was similar when applying the pragmatic threshold of $TBR_{max} \geq 2.0$.

Identical analyses were also performed to assess data-driven thresholds using Youden's index for SUV_{max} thresholds. For carotid imaging, the Youden-derived threshold was 2.41, yielding sensitivity of 70%, specificity of 92%, PPV 77% and NPV of 89%. For cardiac imaging, the optimal SUV_{max} threshold was 3.77, yielding sensitivity of 100%, specificity of 82%, PPV 50% and NPV of 100%.

Supplementary Tables

Table S1.
Background blood pool activity and comparison of TBR_{max}, TBR_{mean}, SUV_{max} and SUV_{max} in visually negative and positive regions of interest.

	**[18F]GP1 Quantification**		
**Mean Background Blood Pool Activity (SUVmean)**			
Internal Jugular Vein (carotid artery)	1·66±0·47		
Right Atrium (valvular and left atrial appendage)	2·12±0·69		
Contralateral Brain Tissue (brain)	0·32±0·27		
**Median TBR_{max} Region of Interest**	**Qualitatively Positive**	**Qualitatively Negative**	**P value**
Carotid Artery TBR_{max}	1·78 [1·57 to 2·51]	1·11 [1·03 to 1·18]	**<0·001**
Left Atrial Appendage TBR_{max}	2·12 [2·09 to 3·51]	1·05 [0·98 to 1·09]	**<0·001**
Brain TBR_{max}	6·46 [6·46 to 12·42]	1·09 [0·66 to 1·33]	**<0·001**
Valvular TBR_{max}	3·69 [2·68 to 4·25]	1·42 [1·18 to 1·67]	**<0·001**
**Median TBR_{mean} Region of Interest**	**Qualitatively Positive**	**Qualitatively Negative**	**P value**
Carotid Artery TBR_{mean}	1·24 [1·07 to 1·72]	0·88 [0·73 to 1·00]	**<0·001**
Left Atrial Appendage TBR_{mean}	1·47 [1·35 to 1·68]	1·03 [0·98 to 1·09]	**<0·001**
Brain TBR_{mean}	2·40 [1·62 to 4·24]	0·99 [0·90 to 1·08]	**<0·001**
Valvular TBR_{mean}	1·90 [1·52 to 2·22]	1·00 [0·93 to 1·12]	**<0·001**
**Median SUV_{max} Region of Interest**	**Qualitatively Positive**	**Qualitatively Negative**	**P value**
Carotid Artery SUV_{max}	2·87 [2·19 to 3·58]	1·85 [1·53 to 2·15]	**<0·001**
Left Atrial Appendage SUV_{max}	3·77 [3·50 to 3·86]	2·24 [1·77 to 2·62]	**<0·001**
Brain SUV_{max}	2·08 [1·22 to 3·01]	0·34 [0·20 to 0·42]	**<0·001**
Valvular SUV_{max}	4·81 [4·66 to 9·99]	2·94 [2·59 to 3·38]	**<0·001**
**Median SUV_{mean} Region of Interest**	**Qualitatively Positive**	**Qualitatively Negative**	**P value**
Carotid Artery SUV_{mean}	1·99 [1·59 to 2·40]	1·47 [1·18 to 1·74]	**<0·001**
Left Atrial Appendage SUV_{mean}	2·20 [1·66 to 2·51]	1·25 [0·87 to 1·63]	**0·006**
Brain SUV_{mean}	1·19 [0·77 to 1·65]	0·39 [0·31 to 0·51]	**<0·001**

Valvular SUV_{mean}	2·69 [2·36 to 3·10]	2·10 [1·78 to 2·48]	**<0·001**

Mean±standard deviation, median [interquartile interval]
Mann-Whitney test was used to determine p values

Table S2.
Intra- and inter-reader quantitative [^{18}F]GP1 reproducibility.

	Intraclass Correlation Coefficient (95% Confidence Interval)	Mean Of Differences (Limits Of Agreement)
**Left carotid intra-observer**		
TBR_{max}	0·887 (0·836 to 0·923)	-0·007 (-0·504 to 0·490)
SUV_{max}	0·851 (0·786 to 0·897)	-0·018 (-0·806 to 0·769)
TBR_{mean}	0·903 (0·860 to 0·934)	-0·028 (-0·403 to 0·347)
SUV_{mean}	0·857 (0·795 to 0·902)	-0·030 (-0·621 to 0·562)
**Right carotid intra-observer**		
TBR_{max}	0·916 (0·878 to 0·943)	-0·027 (-0·562 to 0·507)
SUV_{max}	0·896 (0·849 to 0·929)	-0·023 (-0·845 to 0·799)
TBR_{mean}	0·883 (0·831 to 0·920)	0·023 (-0·347 to 0·392)
SUV_{mean}	0·869 (0·812 to 0·910)	0·032 (-0·551 to 0·614)
**Left carotid inter-observer**		
TBR_{max}	0·814 (0·723 to 0·876)	-0·072 (-0·565 to 0·422)
SUV_{max}	0·899 (0·851 to 0·932)	-0·041 (-0·583 to 0·501)
TBR_{mean}	0·810 (0·680 to 0·883)	-0·095 (-0·520 to 0·330)
SUV_{mean}	0·850 (0·777 to 0·900)	-0·071 (-0·609 to 0·466)
**Right carotid inter-observer**		
TBR_{max}	0·871 (0·804 to 0·916)	-0·081 (-0·641 to 0·476)
SUV_{max}	0·893 (0·842 to 0·928)	-0·023 (-0·667 to 0·621)
TBR_{mean}	0·818 (0·696 to 0·888)	-0·108 (-0·604 to 0·388)
SUV_{mean}	0·878 (0·815 to 0·920)	-0·073 (-0·586 to 0·440)
	**Intraclass Correlation Coefficient (95% Confidence Interval)**	**Mean Of Differences (Limits Of Agreement)**
**Aortic valve intra-observer**		
TBR_{max}	0·998 (0·996 to 0·999)	0·001 (-0·088 to 0·090)
SUV_{max}	0·997 (0·995 to 0·999)	0·006 (-0·209 to 0·221)
TBR_{mean}	0·994 (0·989 to 0·997)	-0·001 (-0·053 to 0·050)
SUV_{mean}	0·993(0·987 to 0·997)	0·006 (-0·209 to 0·221)
**Aortic valve inter-observer**		
TBR_{max}	0·970 (0·938 to 0·985)	0·060 (-0·283 to 0·403)
SUV_{max}	0·954 (0·768 to 0·984)	0·305 (-0·392 to 1·00)
TBR_{mean}	0·868 (0·752 to 0·932)	0·002 (-0·251 to 0·254)
SUV_{mean}	0·907 (0·799 to 0·955)	0·112 (-0·371 to 0·595)
**Mitral valve intra-observer**		
TBR_{max}	0·987 (0·976 to 0·993)	0·020 (-0·145 to 0·186)
SUV_{max}	0·995 (0·990 to 0·997)	0·007 (-0·171 to 0·184)
TBR_{mean}	0·983 (0·968 to 0·991)	0·015 (-0·094 to 0·125)
SUV_{mean}	0·992 (0·985 to 0·996)	0·009 (-0·143 to 0·161)
**Mitral valve inter-observer**		
TBR_{max}	0·979 (0·960 to 0·989)	-0·013 (-0·199 to 0·173)
SUV_{max}	0·975 (0·954 to 0·987)	-0·041 (-0·418 to 0·336)
TBR_{mean}	0·923 (0·860 to 0·959)	-0·001 (-0·178 to 0·176)
SUV_{mean}	0·971 (0·947 to 0·985)	-0·014 (-0·281 to 0·253)

Table S3.
Qualitative and quantitative [^{18}F]GP1 cardiovascular and brain parenchymal uptake stratified by time from stroke symptom onset to time of [^{18}F]GP1 PET/CT scan.

Time from Stroke Symptom Onset to [^{18}F]GP1 PET/CT Scan (days)	Number of patients	Qualitative [^{18}F]GP1 uptake (%)	Quantitative [^{18}F]GP1 uptake TBR_{max}
**Cardiovascular Uptake**			
≤7 days	12	7 (58)	2·01±0·75
8 to 14 days	48	33 (69)	1·96± 0·56
>14 to 21 days	40	23 (58)	1·93±0·61
**Brain Uptake**			
≤7 days	12	10 (83)	15·00±12·55
8–14 days	48	39 (81)	10·04±9·88
>14 to 21 days	40	34 (85)	9·35±6·49

Mean±standard deviation, n (%)

Table S4.
Baseline characteristics in patients with and without cardiovascular [18F]GP1 uptake

**Baseline Characteristics**	**No [18F]GP1 uptake n=37**	**[18F]GP1 uptake n=63**	**P value**
Age (years)	65 [54 to 73]	67 [56 to 73]	0·432
Male	22 (59)	46 (73)	0·238
Systolic Blood Pressure (mmHg)	139 [124 to 148]	143 [126 to 163]	0·565
Diastolic Blood Pressure (mmHg)	81 [75 to 88]	80 [74 to 89]	0·797
Body Mass index (kg/m^2)	30·3±7·8	29·4±7·9	0·587
Smoking Status			0·233
Never	20 (54)	35 (56)	
Current	12 (32)	11 (17)	
Ex-smoker	5 (14)	17 (27)	
**Comorbidities and risk factors**			
Previous Stroke or TIA	1 (3)	12 (19)	**0·041**
Family history of Stroke or TIA	14 (38)	14 (22)	0·147
Hypertension	17 (46)	39 (62)	0·179
Hypercholesterolemia	15 (41)	34 (54)	0·276
Diabetes Mellitus	7 (19)	14 (22)	0·891
History of Atrial Fibrillation or Flutter	2 (5)	7 (11)	0·548
Prosthetic Valve	0	4 (6)	0·142
Ischemic Heart Disease	3 (8)	7 (11)	0·520
Peripheral Vascular disease	0 (0)	2 (3)	0·723
Left ventricular impairment (Ejection Fraction <55%)	1 (3)	7 (11)	0·265
New Atrial fibrillation on Presentation	0 (0)	5 (8)	0·646
Patent Foramen Ovale	11 (30)	13 (21)	0·247
**Medications***			
Dual Antiplatelet Therapy	25 (68)	32 (51)	0·154
Anticoagulation	2 (5)	17 (27)	**0·017**
**Biomarkers**			
Hemoglobin (g/L)	146 ±25	144±14	0·609
Platelets (x10^{9}cells/L)	273±57	257±75	0·290
Total Cholesterol (mmol/L)	4·3±1·7	4·4±1·4	
High-density lipoprotein (mmol/L)	1·4±0·6	1·4±1·1	0·828
Low-density lipoprotein (mmol/L)	2·3 ±1·2	2·0 ±0·9	0·180
Triglyceride (mmol/L)	1·6±0·9	2·0±2·3	0·368
ESR (mm/hour)	10±8	14±16	0·305
Random Glucose (mmol/L)	7·5±5·5	6·9±3·5	0·579
Fibrinogen (g/L)	3·7±1·4	3·6±0·8	0·830
**Brain Imaging Findings (CT or**			
Cortical Infarction	26 (70)	51 (81)	0·327
Subcortical Infarction	11 (30)	12 (19)	0·327
**Brain Parenchymal [18F] GP1**	27 (73)	54 (86)	0·192
**Stroke Treatment**			
Thrombolysis	3 (8)	5 (8)	>0·999
Thrombectomy	0 (0)	2 (3)	0·723

**Stroke Classification as per Causative Classification System**			0·124
Cardioembolic	12 (32)	21 (33)	
Supra-Aortic Large Artery	2 (5)	7 (11)	
Small Artery Occlusion	9 (24)	5 (8)	
Undetermined cause	14 (38)	27 (43)	
Other uncommon cause†	0 (0)	3 (5)	
**National Institutes of Health Stroke Scale**	3 [1 to 4]	2 [1 to 3]	0·206
**Territory of Infarction**			0·164
>1 Vasculature Territory	2 (5)	6 (10)	
Cerebellum	1 (3)	2 (3)	
Cortex	18 (49)	25 (40)	
Posterior	6 (16)	22 (35)	
Subcortical	10 (27)	8 (13)	
**Time from Symptom Onset to CT/PET** (days)	14 [10 to 18]	13 [10 to 17]	0·714

Mean±standard deviation, n (%), median [interquartile interval]
TIA, Transient ischemic attack; CT, Computed Tomography; MRI, Magnetic Resonance Imaging; PET, Positron Emission Tomography
*Medications at time of scan
†Represents cases of arterial dissection

Table S5·
Baseline characteristics stratified by brain parenchymal [18F]GP1 uptake

Characteristic	No Brain [18F]GP1 Uptake n=19	Brain [18F]GP1 Uptake n=81	p value
Age (years)	69 [58 to 76]	65 [54 to 72]	0·453
Sex (male)	12 (63)	56 (69)	0·818
Systolic Blood Pressure (mmHg)	147 [131 to 156]	139 [124 to 59]	0·222
Diastolic Blood Pressure (mmHg)	78 [75 to 85]	81 [72 to 89]	0·755
Body Mass Index (kg/m^2)	28·7±6·2	30·0±8·2	0·502
Smoking Status			
Never Smoker	12 (63)	43 (53)	
Current Smoker	4 (21)	19 (24)	
Ex-smoker	3 (16)	19 (24)	
**Comorbidities and Risk Factors**			
History of Atrial Fibrillation or Flutter	1 (5)	8 (10)	0.680
Previous Stroke or TIA	1 (5)	12 (15)	0·462
Hypertension	11 (58)	45 (56)	0.906
Hypercholesterolemia	7 (37)	42 (52)	0·261
Diabetes Mellitus	3 (16)	18 (22)	0·753
Ischemic Heart Disease	1 (5)	9 (11)	0·684
Peripheral Artery Disease	0 (0)	2 (3)	>0.999
Family History of Stroke or TIA	8 (42)	20 (25)	0·246
Left Ventricular Systolic Dysfunction*	1 (5)	7 (9)	>0·999
New Atrial fibrillation on Presentation	0 (0)	5 (6)	0·581
Left Atrial Appendage Thrombus	0 (0)	4 (5)	0·734
Left Ventricular Thrombus	0 (0)	2 (3)	>0·999
Prosthetic Valve	0 (0)	4 (5)	0·576
Patent Foramen Ovale	5 (26)	20 (25)	>0.999
**Stroke Classification as per CCS**			**0·041**
Cardioembolic	5 (27)	28 (35)	
Large Artery Atherosclerosis	0 (0)	9 (11)	
Small Artery Occlusion	7 (37)	7 (9)	
Undetermined cause	7 (37)	34 (43)	
Other uncommon cause†	0 (0)	3 (4)	
**Medications****			
Dual Antiplatelet Therapy	14 (74)	43 (53)	0·133
Anticoagulation	1 (5)	18 (22)	0·068
**Time from symptom onset to PET scan** (days)	15 [13 to18]	12 [9 to 17]	0·084
**National Institutes of Health Stroke Scale**	2 [1 to 3]	2 [1 to 4]	0·372
**Sites of [18F] GP1 Uptake**			
Cardiac or Vascular	9 (48)	54 (67)	0·192
Native Valve	1 (5)	10 (12)	0·631
Prosthetic Valve	0 (0)	4 (5)	0·671
Carotid Artery	5 (26)	37 (46)	0·232
Vertebral Artery	1 (5)	9 (11)	0·734
Aorta Arch or Ascending Aorta	2 (10)	2 (3)	0·336

Left Atrium	0 (0)	3 (4)	0·917
**Stroke Treatment**			
Thrombolysis	0 (0)	8 (10)	0·338
Thrombectomy	0 (0)	2 (3)	>0·999
**Territory of infarction**			0·164
>1 Territory	3 (16)	5 (6)	
Cerebellum	1 (5)	2 (3)	
Cortex	5 (26)	38 (47)	
Posterior	4 (21)	24 (30)	
Subcortical	6 (32)	12 (15)	
**Brain Imaging (CT or MRI)**			
Cortical Infarction	9 (47)	68 (84)	**0·002**
Subcortical Infarction	10 (53)	12 (15)	**0·002**
Infarct seen on MRI alone	12 (63)	19 (23)	**0·002**

CT, Computed Tomography; MRI, Magnetic Resonance Imaging; PET, Positron Emission Tomography
n (%), median [interquartile interval], mean±standard deviation
*Left ventricular ejection fraction <55%
**Medications at time of scan*

Table S6.
Degree of Carotid Stenosis With and Without [^{18}F]GP1 Uptake

**Degree of carotid stenosis***	**Number of patients**	**No [^{18}F]GP1 uptake n=57**	**[^{18}F]GP1 uptake n=42**
No stenosis	31	28 (90)	3 (10)
<30% stenosis	43	24 (56)	19 (44)
30-49% stenosis	17	3 (18)	14 (82)
50-69% stenosis	5	1 (20)	4 (80)
>70% stenosis	3	1 (33)	2 (67)

n (%)

*As per the North American Symptomatic Carotid Endarterectomy Trial (NASCET) criteria.

One patient had a non-diagnostic CT angiogram and is excluded from this table, resulting in 99 evaluable patients.

Table S7.
Associations With Recurrent Stroke or Transient Ischemic Attack

	**Hazard Ratio (univariable)**	**Hazard Ratio (multivariable model[1])**	**Hazard Ratio (multivariable model[2])**
**Cardiovascular [^{18}F]GP1 uptake**	10·4 (1·35 to 79·40), p=0·024	9·23 (1·15 to 74·1), p=0·036	9·29 (1·20 to 71·8), p=0·033
**Hypertension**	1·72 (0.54 to 5·49), p=0·40	2·16 (0·59 to 7·97), p=0·20	
**Smoker**	1·71 (0·93 to 3·17), p=0·09	1·56 (0·82 to 2·97), p=0·20	
**Diabetes mellitus**	1·41 (0·44 to 4·51), p=0·60	1·82 (0·52 to 6·35), p=0·30	
**Hypercholesterolemia**	0·57 (0·19 to 1·71), p=0·30	0·35 (0·10 to 1·15), p =0·083	
**Carotid stenosis ≥50%**	0·60 (0·08 to 4·60), p=0·60	0·85 (0·10 to 6·97), p=0·90	
**Prior history of stroke or transient ischemic attack**	2·54 (0·70 to 9·23), p=0·20	1·54 (0·38 to 6·19), p=0·50	
**Family history of stroke or transient ischemic attack**	0·44 (0·10 to 1·97), p=0·30		
**Age (years)**	1·03 (0·98 to 1·08), p=0·20		1·03 (0·98 to 1·08), p=0·20
**Sex**	0·48 (0·13 to 1·72), p=0·30		0·43 (0·12 to 1·58), p=0·20

[1]*Multivariable model to explore whether cardiovascular [^{18}F]GP1 uptake remained associated with recurrent cerebrovascular events after adjustment for conventional vascular risk factors, including hypertension, smoking status, diabetes mellitus, hypercholesterolemia, carotid stenosis ≥50%, and prior history of stroke or transient ischemic attack*

Supplementary Figures

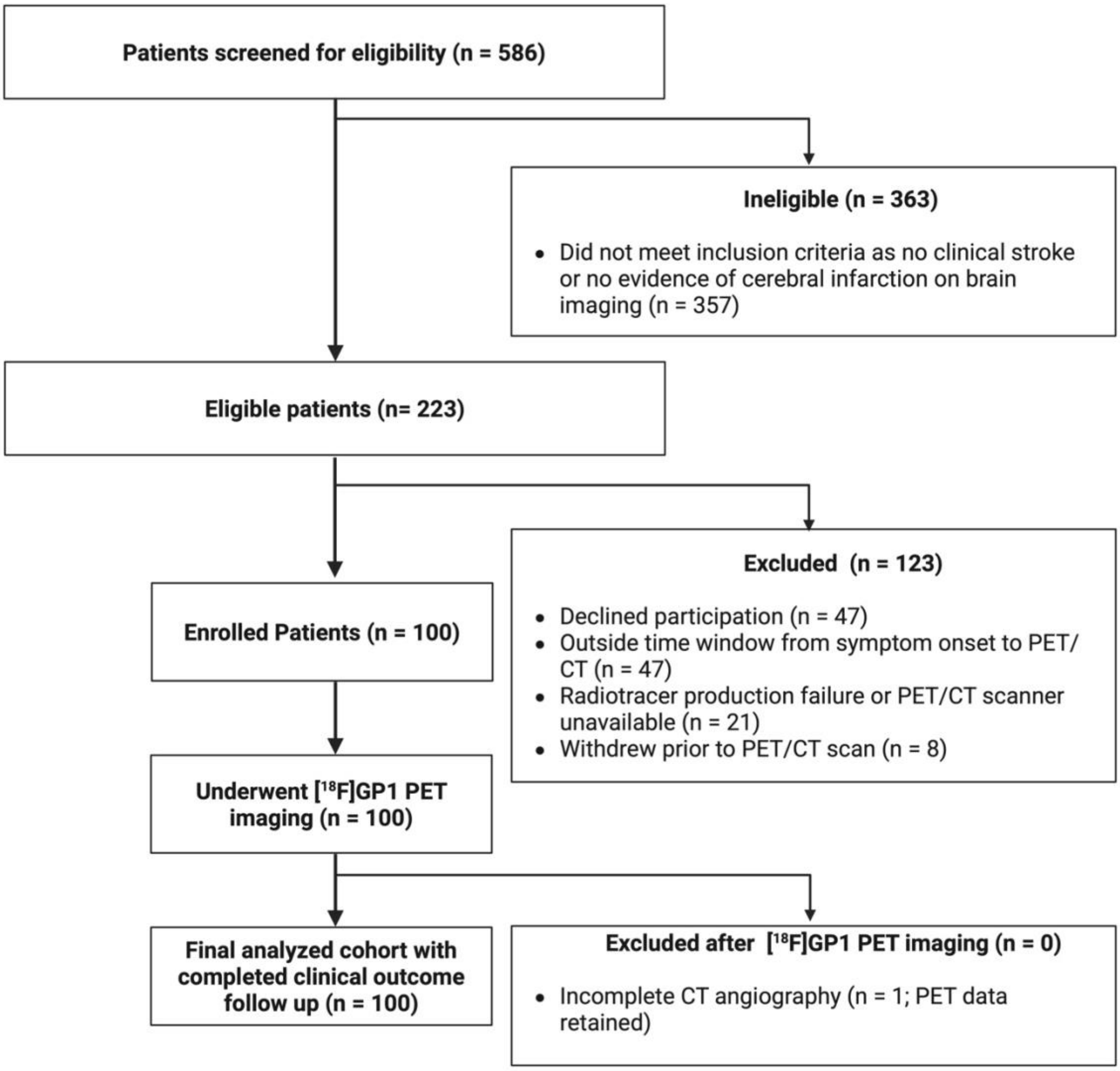

Figure S1. Study flow diagram demonstrating the flow of participants through screening, enrollment, imaging, and final analysis.

A total of 586 patients were assessed for eligibility, of whom 486 were excluded for predefined clinical, logistical, or technical reasons. One participant had incomplete CT angiography of the carotid arch and carotid arteries; however, PET imaging data were retained and included in the final analysis. The final analyzed cohort comprised 100 patients with completed clinical outcome follow-up.

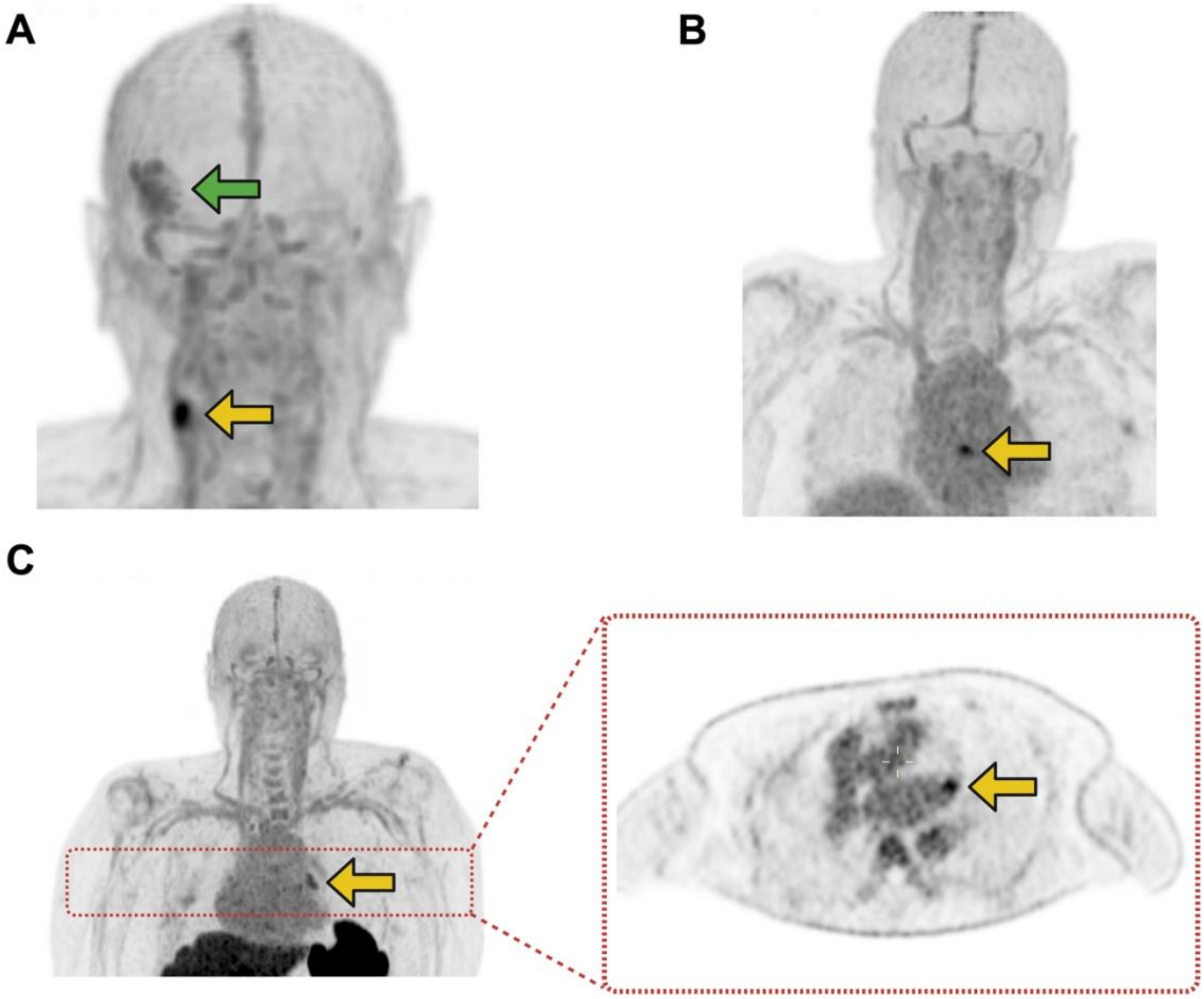

Figure S2. Raw attenuated corrected Fluorine-18 GP1 ([^{18}F]GP1) positron emission tomography images from patients with cardiovascular [^{18}F]GP1uptake.

Panel A (yellow arrow) demonstrating [^{18}F]GP1 uptake in right internal carotid artery suggestive of ipsilateral carotid plaque rupture and thromboembolism and green arrow demonstrating ipsilateral diffuse brain parenchymal [^{18}F]GP1 uptake indicative of brain petechial hemorrhage. Panel B (yellow arrow) showing aortic valve [^{18}F]GP1 uptake in the context of a thickened but non-stenotic native aortic valve as a potential source of cardioembolism. Panel C (yellow arrows) demonstrating [^{18}F]GP1 uptake in the left atrial appendage in a patient with documented history of atrial fibrillation, indicative of left atrial appendage thrombus as a potential cardioembolic source of cerebral infarction.

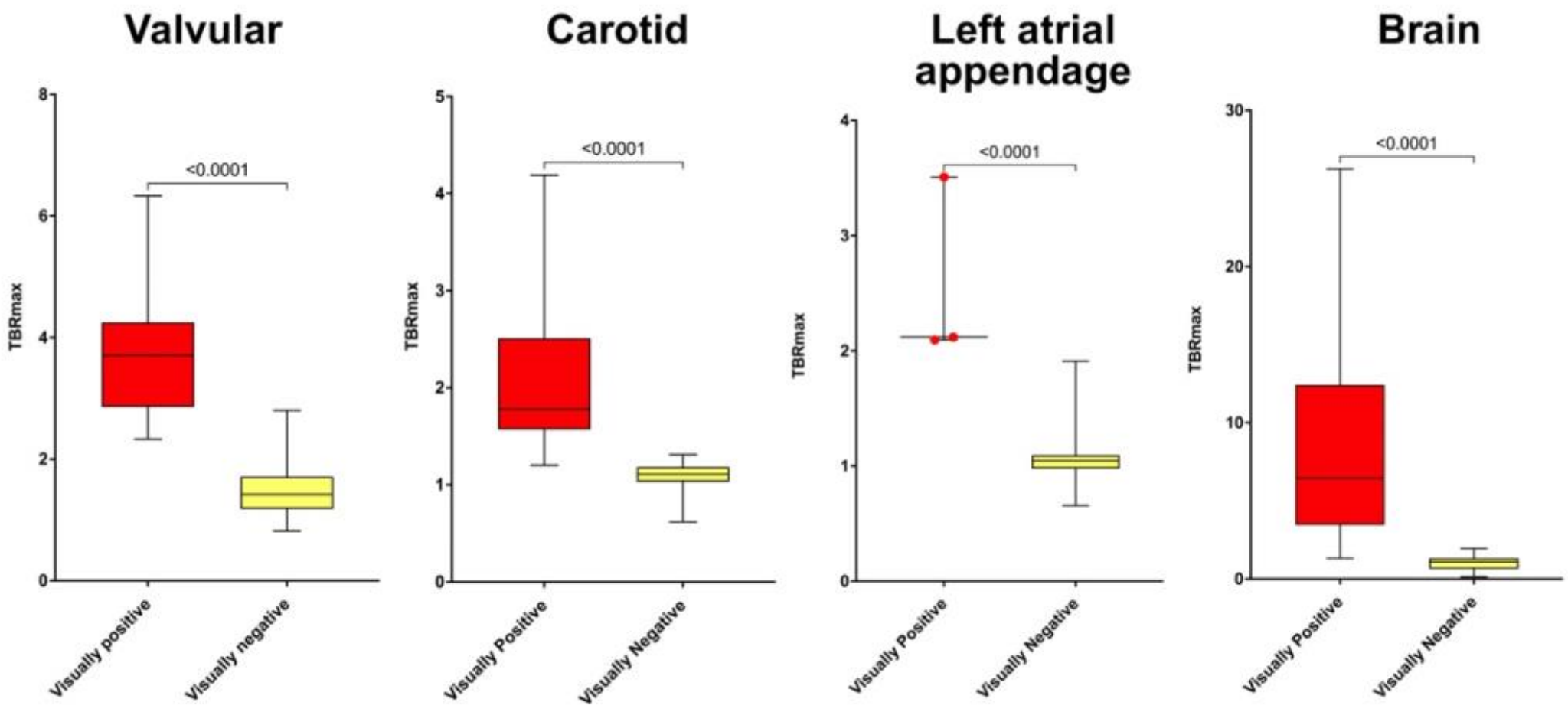

Figure S3. Fluorine-18 GP1 ([18F]GP1) uptake in visually positive and visually negative cardiac valves, carotid arteries, left atrial appendages and brain parenchyma.
Median maximum target to background ratios (TBR_{max}). Median (line), interquartile interval (box) and 95% confidence intervals (bars).

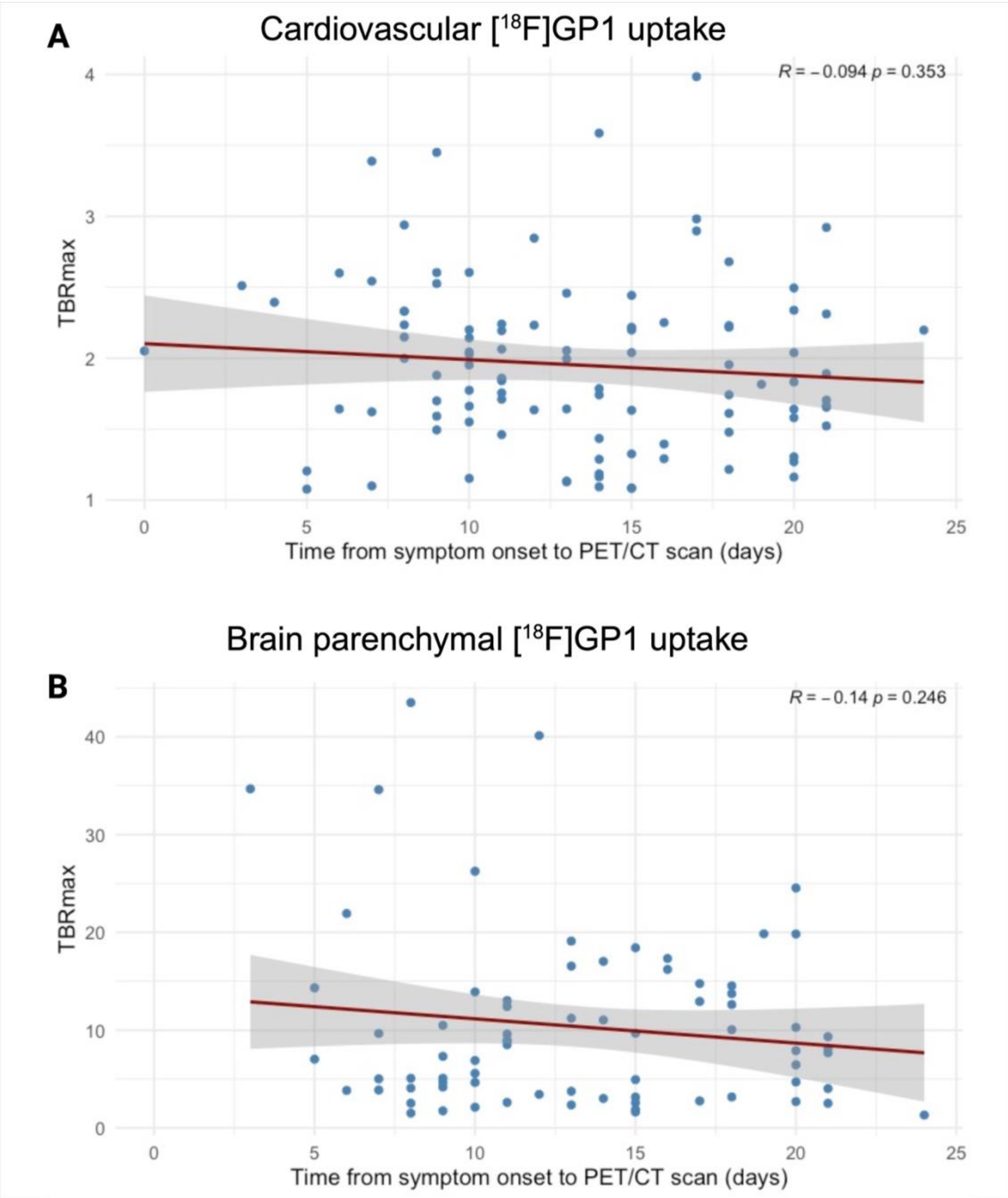

Figure S4. Comparison between time from symptom onset to positron emission tomography and computed tomography (PET/CT) scan and [^{18}F]GP1 uptake.
(**A**) Cardiovascular uptake (R = −0·094, p = 0·353).
(**B**) Brain parenchymal uptake (R = −0·140, p = 0·246)
Red lines indicate linear regression fits with 95% confidence intervals.

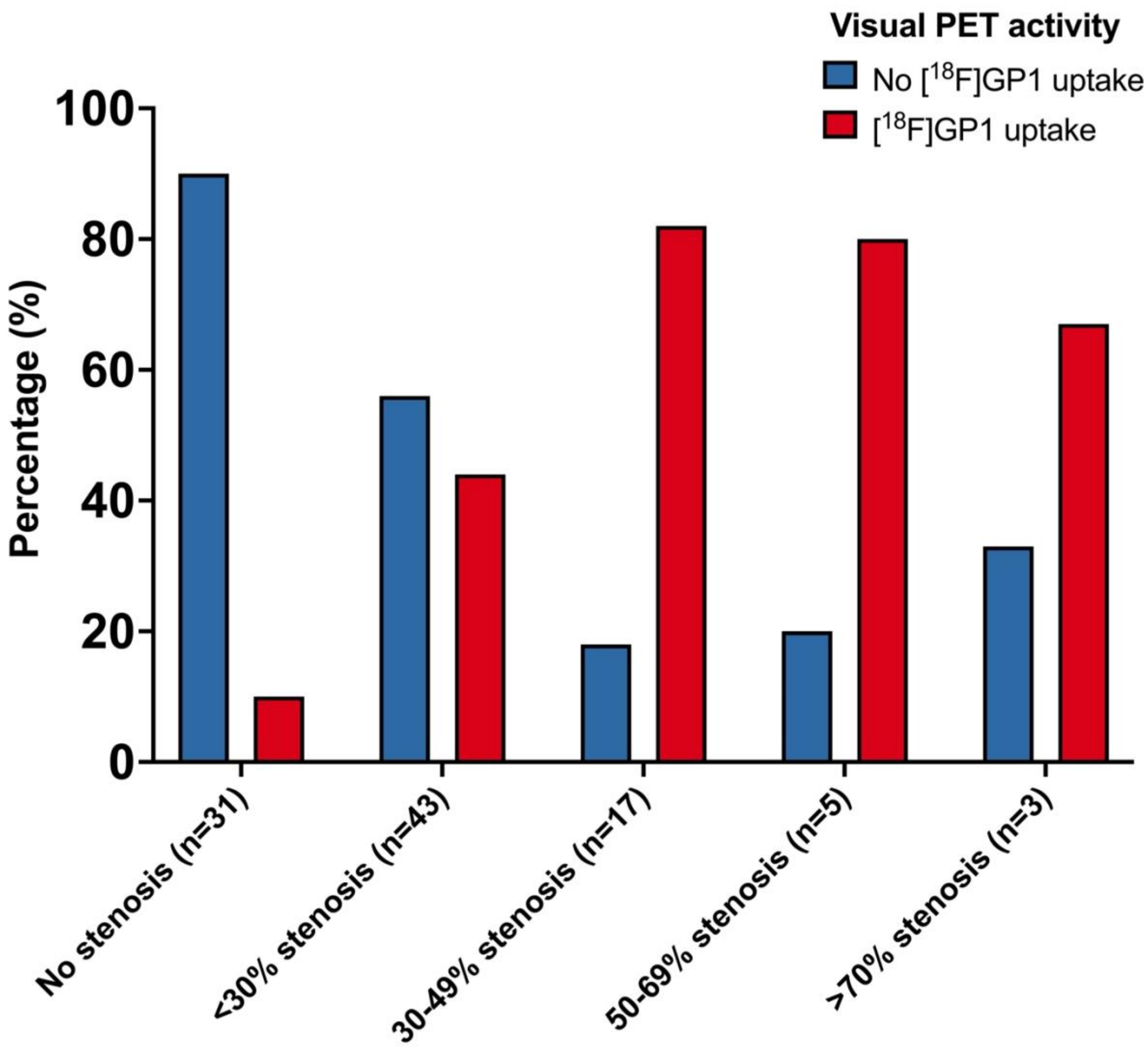

Figure S5. Percentage of patients with and without carotid [^{18}F]GP1 uptake by degree of carotid artery stenosis.

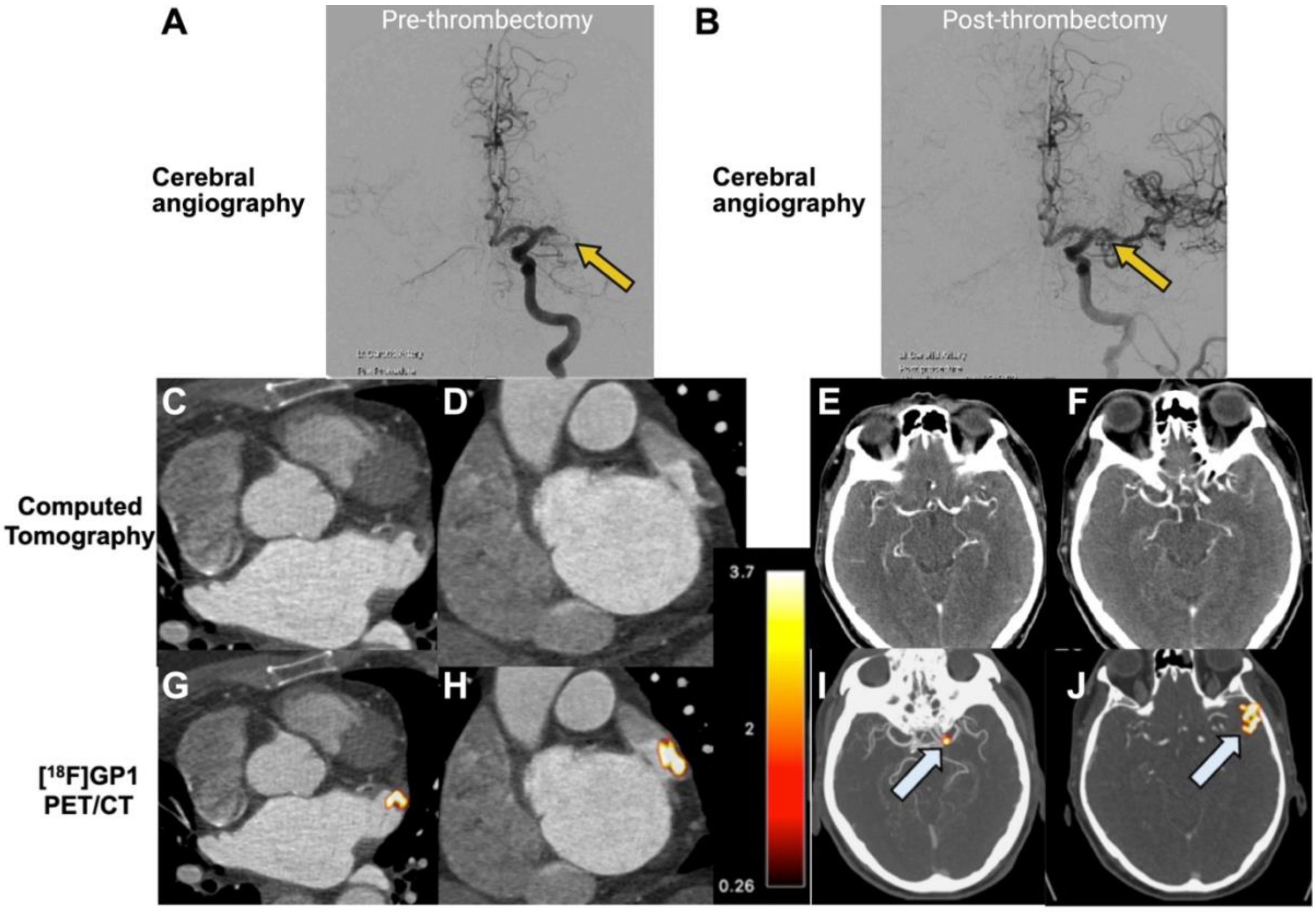

Figure S6. Middle Cerebral Artery Occlusion in Patient with Ischemic Stroke and Atrial Fibrillation. Invasive angiography and fluorine-18 GP1 ([^{18}F]GP1) positron emission tomography and computed tomography (PET/CT) angiography in a patient presenting with right sided weakness and dysphasia and new atrial fibrillation. The patient was treated with thrombectomy with pre (Panel A) and post (Panel B) cerebral angiography showing acute left sided middle cerebral artery occlusion and subsequent resolution of perfusion respectively. Left atrial appendage thrombus was seen on CT angiography (Panels C and D) with [^{18}F]GP1 uptake (Panels G and H). There was also demonstrable [^{18}F]GP1 uptake at site of thrombectomy in the middle cerebral artery (Panel I) and area of infarction (Panel J).
