# Supplementary material for: Noninvasive Thrombus Imaging in Patients With Ischemic Stroke
Source: Circulation. 2026 Sep 1;154(15):1424–40. doi: 10.1161/CIRCULATIONAHA.126.080420 (PMC13668038; doi:10.1161/CIRCULATIONAHA.126.080420)
Supplement: Supplementary file 2 [file cir-154-1424-s002.docx]

STROBE Statement—Checklist of items that should be included in reports of ***cohort studies***

|  | Item No | Recommendation |
| --- | --- | --- |
| **Title and abstract** | 1 | (*a*) Indicate the study’s design with a commonly used term in the title or the abstract  *Addressed in Abstract and Methods section, pages 2 and 7.* |
|  |  | (*b*) Provide in the abstract an informative and balanced summary of what was done and what was found *Page 2.* |
| Introduction | | |
| Background/rationale | 2 | Explain the scientific background and rationale for the investigation being reported  *Introduction section, pages 4 and 5.* |
| Objectives | 3 | State specific objectives, including any prespecified hypotheses  *Addressed in the Introduction section, pages 5 and 6.* |
| Methods | | |
| Study design | 4 | Present key elements of study design early in the paper *Addressed in Methods section, page 7.* |
| Setting | 5 | Describe the setting, locations, and relevant dates, including periods of recruitment, exposure, follow-up, and data collection  *Addressed in Methods section, page 7.* |
| Participants | 6 | (*a*) Give the eligibility criteria, and the sources and methods of selection of participants. Describe methods of follow-up  *Addressed in Methods section, page 7 and in Supplementary Figure 1* |
|  |  | (*b*) For matched studies, give matching criteria and number of exposed and unexposed  *Not applicable.* |
| Variables | 7 | Clearly define all outcomes, exposures, predictors, potential confounders, and effect modifiers. Give diagnostic criteria, if applicable  *Addressed in Methods section pages 7 to 13.* |
| Data sources/ measurement | 8* | For each variable of interest, give sources of data and details of methods of assessment (measurement). Describe comparability of assessment methods if there is more than one group  *Addressed in Methods section, pages 7 to 13.* |
| Bias | 9 | Describe any efforts to address potential sources of bias  *Addressed in Methods section, page 7 to 13.* |
| Study size | 10 | Explain how the study size was arrived at  *The study was designed to be mostly descriptive. The overall sample size was chosen to allow us to explore clinical characteristics of interest and a number of exploratory analyses.* *For 80% power at two-sided p<0.05 to detect correlations of r²≥0.05, required a target sample size of 120. For 120 patients, we estimated ≥95% power at two-sided P<0.05 to detect the occurrence of thrombosis of >5% for cryptogenic stroke. Recruitment was conducted over a predefined period (March 2023 to June 2025), with 100 patients enrolled in the final cohort.* |
| Quantitative variables | 11 | Explain how quantitative variables were handled in the analyses. If applicable, describe which groupings were chosen and why  *Addressed in Methods section page 12 and 13.* |
| Statistical methods | 12 | (*a*) Describe all statistical methods, including those used to control for confounding  *Addressed in Methods section page 12 and 13.* |
|  |  | (*b*) Describe any methods used to examine subgroups and interactions  *Not applicable.* |
|  |  | (*c*) Explain how missing data were addressed  *Missing data were minimal. No imputation was performed.* |
|  |  | (*d*) If applicable, explain how loss to follow-up was addressed  *Complete follow-up was achieved for all 100 participants.* |
|  |  | (*e*) Describe any sensitivity analyses  *Multivariable Cox regression models adjusting for conventional vascular risk factors are presented in Supplementary Table 7. Age and sex were included in a separate sensitivity model. now* |
| Results | | |
| Participants | 13* | (a) Report numbers of individuals at each stage of study—eg numbers potentially eligible, examined for eligibility, confirmed eligible, included in the study, completing follow-up, and analysed  *Addressed in Supplementary figure 1.* |
|  |  | (b) Give reasons for non-participation at each stage  *Addressed in Supplementary figure 1.* |
|  |  | (c) Consider use of a flow diagram  *Addressed in Supplementary figure 1.* |
| Descriptive data | 14* | (a) Give characteristics of study participants (eg demographic, clinical, social) and information on exposures and potential confounders *Addressed in table 1.* |
|  |  | (b) Indicate number of participants with missing data for each variable of interest  *Missing data were minimal across all variables of interest and are indicated in Table 1 where applicable.* |
|  |  | (c) Summarise follow-up time (eg, average and total amount)  *Addressed in Results page 19.* |
| Outcome data | 15* | Report numbers of outcome events or summary measures over time  *Addressed in Results page 14 to 20.* |
| Main results | 16 | (*a*) Give unadjusted estimates and, if applicable, confounder-adjusted estimates and their precision (eg, 95% confidence interval). Make clear which confounders were adjusted for and why they were included. *Addressed in Results page 14 to 20, table 2 and supplementary table 2.* |
|  |  | (*b*) Report category boundaries when continuous variables were categorized  *Not applicable.* |
|  |  | (*c*) If relevant, consider translating estimates of relative risk into absolute risk for a meaningful time period  *Not applicable.* |
| Other analyses | 17 | Report other analyses done—eg analyses of subgroups and interactions, and sensitivity analyses *Exploratory receiver operating characteristic (ROC) analyses were performed to assess data-driven quantitative thresholds for [^18^F]GP1 uptake using Youden’s index across carotid and cardiac vascular territories. Results are presented in Supplementary Methods and Supplementary Tables 3 and 4.* |
| Discussion | | |
| Key results | 18 | Summarise key results with reference to study objectives  *Addressed in the Discussion section page 21.* |
| Limitations | 19 | Discuss limitations of the study, taking into account sources of potential bias or imprecision. Discuss both direction and magnitude of any potential bias  *Addressed in the Discussion section page 25.* |
| Interpretation | 20 | Give a cautious overall interpretation of results considering objectives, limitations, multiplicity of analyses, results from similar studies, and other relevant evidence  *Addressed in the Discussion section page 26 to 27.* |
| Generalisability | 21 | Discuss the generalisability (external validity) of the study results  *Addressed in the Discussion section page 26 to 27.* |
| Other information | | |
| Funding | 22 | Give the source of funding and the role of the funders for the present study and, if applicable, for the original study on which the present article is based  *Addressed in the Acknowledgments page 28.* |

*Give information separately for exposed and unexposed groups.

**Note:** An Explanation and Elaboration article discusses each checklist item and gives methodological background and published examples of transparent reporting. The STROBE checklist is best used in conjunction with this article (freely available on the Web sites of PLoS Medicine at http://www.plosmedicine.org/, Annals of Internal Medicine at http://www.annals.org/, and Epidemiology at http://www.epidem.com/). Information on the STROBE Initiative is available at http://www.strobe-statement.org.
